# Endothelial glycocalyx perturbation in obstructive sleep apnea is associated with repetitive hypoxemia and immunothrombotic endothelial dysfunction

**DOI:** 10.1186/s12967-026-08409-2

**Published:** 2026-06-12

**Authors:** Martin Bernhard Müller, Tobias Kammerer, Humayun Khan, Annika Schmid, Simon Hirschberger, Max Hübner, Teresa K. Barth, Rea Mitsigiorgi, Clemens Stihl, Martin Holzer, Daniel Jira, Martin Patscheider, Bernhard G. Weiss, Christoph Andreas Reichel, Bernd Uhl

**Affiliations:** 1 Department of Anaesthesiology, LMU University Hospital, Ludwig-Maximilians-Universität München (LMU), Munich, Germany; 2 Walter Brendel Center of Experimental Medicine, Ludwig-Maximilians-Universität München (LMU), Munich, Germany; 3 Protein Analysis Unit, BioMedical Center, Faculty of Medicine, Ludwig-Maximilians-Universität München (LMU), Martinsried, Germany; 4 Department of Otorhinolaryngology, LMU University Hospital, Ludwig-Maximilians-Universität München (LMU), Munich, Germany; 5 Department of Otorhinolaryngology, Head and Neck Surgery, School of Medicine and Health, TUM University Hospital, Technical University of Munich, Munich, Germany; 6 Comprehensive Cancer Center Munich, Ludwig-Maximilians-Universität (LMU), LMU University Hospital, Munich, Germany

**Keywords:** Endothelial glycocalyx, Obstructive sleep apnea, Intermittent hypoxia, Hyaluronan, Heparan sulfate, Oxidative stress, Immunothrombosis, Hypoxic burden

## Abstract

**Background:**

Obstructive sleep apnea (OSA) is associated with cardiovascular disease. The endothelial glycocalyx (eGCX) is a shear-sensitive intravascular barrier. The relevance of OSA and intermittent hypoxia (IH) for eGCX perturbation and cardiovascular disease in humans remains unclear.

**Methods:**

In a prospectively recruited observational cohort with cross-sectional biomarker analysis in men (*n* = 60), polysomnography quantified apnea-hypopnea index (AHI), oxygen desaturation index (ODI), and hypoxic burden (HB). Using single-time-point plasma sampling, eGCX glycosaminoglycans hyaluronan (HA) and heparan sulfate (HS) and the proteoglycan syndecan-1 (SDC-1) were related to OSA severity and repetitive hypoxemia, including multivariable adjustment for cardiometabolic, inflammatory, and renal determinants. Plasma proteomics defined pathways associated with OSA and HA/HS. Shear-matured primary human endothelial cells were exposed to OSA-characteristic IH cycles under arterial flow to assess eGCX structure, oxidative stress, nitric oxide (NO) signaling, thromboinflammatory status, and antioxidant treatment with N-acetylcysteine.

**Results:**

Plasma HA and HS were higher in OSA than in non-OSA individuals and increased stepwise with disease severity, tracking AHI, ODI, and HB. These associations persisted in multivariable analyses adjusting for age, BMI, hypertension, hs-CRP, fasting glucose, and eGFR. Plasma SDC-1 did not differ between groups and remained non-associated in adjusted analyses. Proteomics revealed enrichment of inflammatory, coagulation, and oxidative stress-related pathways that strengthened with increasing OSA burden and higher HA/HS levels. Experimentally, IH caused loss of endothelial surface HA/HS with increased shedding, increased reactive oxygen species, reduced redox capacity, impaired NOS3/eNOS signaling and reduced NO bioavailability. IH or enzymatic eGCX digestion each enhanced monocyte and platelet adhesion, tissue factor expression, and fibrin deposition under shear, while N-acetylcysteine attenuated oxidative stress and partially restored surface HA expression.

**Conclusions:**

Integrated patient and IH-model data show that OSA severity and repetitive hypoxemia are associated with circulating markers consistent with eGCX perturbation, while the endothelial IH model supports induction of oxidative-inflammatory stress and a proadhesive, prothrombotic phenotype by intermittent hypoxia. Together, circulating HA and HS emerge as candidate biomarkers associated with OSA-related eGCX perturbation, warranting further evaluation in longitudinal and interventional studies of eGCX-stabilizing adjunct therapies. These findings derive from an all-male cohort and require validation in women and more diverse populations.

**Supplementary Information:**

The online version contains supplementary material available at 10.1186/s12967-026-08409-2.

## Background

Obstructive sleep apnea (OSA) affects roughly one billion people worldwide and is tightly linked to cardiovascular diseases (CVD) beyond sleep-related symptoms [1, 2]. The hallmark of OSA pathophysiology is recurrent upper airway obstruction, which results in intermittent hypoxia (IH). Depth, duration, and frequency of desaturations as measured by oxygen desaturation index (ODI) and hypoxic burden (HB) correlate with the OSA-associated vascular risk and CVD mortality [3–6]. This highlights hypoxic burden as a key driver of cardiovascular complications.

Although meta-analyses suggest that continuous positive airway pressure (CPAP) might principally lower all-cause and cardiovascular mortality of OSA, a large, randomized trial revealed no reduction in major cardiovascular events, emphasizing the need for mechanism-targeted adjunctive strategies and improved phenotyping [7, 8]. Accordingly, a recent multi-trial analysis suggests that therapy with CPAP may be preferentially beneficial for patients with high-risk OSA as characterized by greater OSA-related heart rate acceleration or worse hypoxemia [9].

However, currently used stratification approaches rely largely on physiologic patterns [10] and on circulating surrogate markers of inflammation/oxidative stress or endothelial activation [11–13]. These markers are often non-specific and strongly confounded by comorbidities (e.g., obesity/metabolic disease), which limits their mechanistic interpretability [14]. Altogether, there remains a need for mechanism-anchored biomarkers that capture hypoxemia-related vascular perturbation in OSA [15–17].

OSA-associated intermittent hypoxia promotes endothelial inflammation and oxidative stress and reduces nitric oxide bioavailability, thereby impairing endothelial homeostasis [12, 13, 16]. Inflammatory endothelial activation is known to shift the vascular surface towards a proadhesive and prothrombotic phenotype, providing a plausible mechanistic link between OSA and cardiovascular remodeling [18].

We recently introduced a novel in vitro model of shear-matured endothelial cells exposed to OSA-analogous IH that approximates the frequency and severity of hypoxic events seen in OSA patients [19]. By applying this model, we revealed that classical hypoxic signaling is only modestly triggered by OSA-like IH, whereas inflammatory endothelial activation is pronounced.

A biologically plausible, but insufficiently tested link between OSA and CVD is whether OSA-mediated inflammation affects the endothelial glycocalyx (eGCX). The eGCX is a glycan-rich layer on the cell membrane built of syndecan and glypican core proteins bearing heparan sulfate (HS) and further glycosaminoglycans, together with receptor-bound hyaluronan (HA) [20, 21]. It maintains barrier function towards the intravascular compartment, which includes shear sensing, limiting leukocyte and platelet adhesion, and presenting anti-coagulant glycosaminoglycans that restrain coagulation. Inflammatory and oxidative stress are associated with increased enzymatic eGCX shedding and endothelial dysfunction, supporting glycocalyx disruption as an increasingly recognized contributor to CVD[22–25].

Due to their central structural role in the eGCX, HA and HS are promising candidate circulating biomarkers for clinical phenotyping and future longitudinal/interventional monitoring. Several studies analyzing OSA patients report elevations of glycocalyx (GCX)-related surrogates as indicators for GCX perturbation, including the sheddases heparanase and hyaluronidase-1 [26, 27] as well as the endothelial proteoglycan endocan/ESM-1 [11, 28–31]. However, quantitative data on core eGCX components (circulating HA and HS, or syndecan-1 (SDC-1) shedding) in OSA patients remain scarce and inconsistent [26, 32].

In this study, we combined a cross-sectional biomarker analysis in a prospectively recruited observational cohort with plasma proteomics and a shear-matured endothelial rapid-cycling IH model. This approach allows us to examine clinical associations between OSA severity/repetitive hypoxemia and circulating eGCX markers in humans, while testing the effects of OSA-like IH on endothelial cells in vitro.

## Methods

### Study participants and biosamples

We conducted a prospective, single-center observational study at the interdisciplinary sleep laboratory of the Department of Otorhinolaryngology, University Hospital, LMU Munich (March 2016 – June 2021). All study participants provided written informed consent in accordance with the Declaration of Helsinki. The study protocol and the use of clinical data and biosamples for research were approved by the Institutional Ethics Committee. Eligible participants were men aged > 18 years. The cohort was restricted to men in this primary study a priori in order to reduce potential variability related to menstrual-cycle-associated changes in circulating glycocalyx-related markers [33]. Participants with central sleep apnea, cardiovascular disease, diabetes, autoimmune disease, immunosuppression, advanced malignant disease, severe chronic kidney disease, and/or advanced liver disease were excluded.

We screened 86 individuals and aimed a priori to include 15 participants per AHI-defined stratum (AHI non-OSA: <5; mild OSA: 5–<15; moderate OSA: 15–<30; severe OSA: ≥30 events/h) to obtain balanced group sizes. When more than 15 eligible individuals were available within a stratum, we included the first 15 consecutive participants (chronological order of study enrollment/ID) with available EDTA plasma and complete PSG/oximetry variables; selection was performed independent of biomarker values. An age-comparable non-OSA cohort was recruited from the same setting and recruitment period; age did not differ materially between groups.

After inclusion, venous blood was drawn and EDTA plasma (S-Monovette EDTA K3E, 4.9 ml, Sarstedt, Nümbrecht, Germany) was obtained by centrifugation at 2,000 g for 10 minutes, aliquoted and stored at − 80 °C until analysis. Technicians performing ELISA measurements were blinded to clinical information and group assignment; all assays including proteomics used a single-use plasma aliquot that had not been previously thawed. In-patient laboratory polysomnography was performed, and oximetry data were processed with SleepWork 9 (Natus, Planegg, Germany). In the non-OSA group, *n* = 10 participants were diagnosed using the SOMNOcheck microCARDIO, oximetry data were processed with SOMNOlab 2.11 software (Löwenstein Medical, Bad Ems, Germany). Automated indices and event scores were reviewed and, where necessary, corrected by experienced sleep technicians. The oxygen desaturation index (ODI3%) was defined as the average number of desaturation events with >3% decline in oxygen saturation per hour of sleep, according to the American Academy of Sleep Medicine [34]. Oxygen desaturation area capturing depth and duration of the desaturation curve was measured as hypoxic burden (HB) according to Sutherland et al. [35].

### Clinical variables

Additional clinical covariates for the biomarker analyses were obtained from the routine clinical work-up and standard hospital laboratory measurements at LMU University Hospital. These variables included smoking status, arterial hypertension, high-sensitivity C-reactive protein (hs-CRP), fasting glucose, estimated glomerular filtration rate (eGFR), albumin, and cholinesterase, selected to capture major cardiometabolic, inflammatory, renal, and hepatic determinants potentially influencing circulating HA, HS, and SDC-1 concentrations.

### Endothelial cell culture and shear conditioning

Primary human umbilical vein endothelial cells (HUVECs) were isolated from umbilical cords of healthy neonates immediately after cesarean delivery at the Department of Gynecology and Obstetrics, University Hospital, LMU Munich. Written informed consent was obtained from the mothers in accordance with the Declaration of Helsinki. HUVECs were isolated and cultured as previously described [36]. Non-pooled HUVECs from individual donors were used for independent experiments at passages 2–4.

For shear conditioning, µ-Slides I 0.2 Luer (glass-bottom channel, height 250 µm; ibidi, Gräfelfing, Germany; Cat# 80167) were coated (poly-L-lysine, glutaraldehyde, gelatin) and equilibrated in ECGM. HUVECs were seeded on both sides of the channel to obtain stable monolayers, then connected to an ibidi pump system and cultured overnight under unidirectional flow (5 dyn/cm^2^). Shear stress was increased to 15 dyn/cm^2^ for 48 h to mimic arterial flow conditions [37, 38].

### Rapid-cycling IH under flow

Rapid-cycling IH under flow was applied using a custom two-circuit perfusion system as previously described [19]. µ-Slides I 0.2 Luer with endothelial monolayers seeded on both opposing channel surfaces were perfused at 15 dyn/cm^2^ with either normoxic or pre-degassed hypoxic medium, while pericellular oxygen tension was continuously monitored using a *Licox* probe (Brain Tissue Oxygen Monitoring system, Integra). In the present study, we used two previously validated OSA-like IH paradigms, 5 cycles/h for 4 h and 15 cycles/h for 4 h, which are referred to throughout this manuscript as lower-burden and higher-burden IH, respectively [19]. Representative pericellular oxygen traces for these paradigms are shown in Supplementary Fig. S1 (adapted from Müller et al. [19]).

The two paradigms differed in their pericellular oxygen-cycling profiles and were therefore interpreted as lower- versus higher-burden IH conditions, rather than as a pure cycle-frequency series. In the 15 cycles/h condition, oxygen partial pressure cycled from 80 to 90 mmHg to a nadir of 35 mmHg, with a mean time to nadir of 78 s and a mean time back to peak of 46 s, resulting in rapid repetitive hypoxia–reoxygenation episodes. These IH settings were selected to reflect a higher-burden OSA-like hypoxia pattern under flow. In the 15 cycles/h condition, the nadir of approximately 35 mmHg corresponds to a severe desaturation range previously documented in patients with severe OSA [19]. In the 5 cycles/h protocol, the longer reoxygenation intervals allowed pericellular oxygen tension to return closer to air-equilibrated medium before the subsequent hypoxic drop, resulting in higher peak oxygen values than in the 15 cycles/h protocol. After IH exposure, slides were used for functional assays or cells were harvested for downstream analyses.

### Endothelial monocyte adhesion assay under flow

Primary human monocytes were isolated *via* CD14-MicroBeads (Miltenyi Biotec, Bergisch Gladbach, Germany; Cat#130–096-537) from PBMC stimulated with 25 ng/mL CCL2 (45 min) and labeled with 1 µM calcein (30 min). After flow conditioning ± IH, monocytes were perfused over HUVEC monolayers using stepwise shear (5 dyn/cm^2^ for 45 min; 10 dyn/cm^2^ for 5 min; 20 dyn/cm^2^ for 10 min) as previously described [39]. Fluorescence images were acquired with a confocal microscope (Leica TCS SP5; 10× objective). Firmly adherent monocytes were quantified using ImageJ (NIH, Bethesda, MD, USA) (10 fields/slide).

### Endothelial fibrin deposition assay under flow

Fibrin formation and deposition on endothelial monolayers was assessed using a parallel-plate flow chamber. Perfused components were pre-coated with anti-clot buffer (36 mM citric acid, 103 mM NaCl, 5 mM KCl, 5 mM EDTA, 0.35% BSA, pH 6.5). After IH, slides were perfused with citrated whole blood from healthy donors with inline recalcification (85 mM CaCl_2_ in Tyrode buffer). Blood and CaCl_2_ were co-perfused at 13 mL/h (≈1 dyn/cm^2^), followed by a Tyrode wash (134 mL/h; ≈5 dyn/cm^2^). Slides were fixed and stained for fibrin using an anti-fibrin antibody (clone 59D8; MilliporeSigma, Burlington, MA, USA; Cat# MABS2155-25UG; 1:250) followed by an Alexa Fluor 488-conjugated anti-mouse IgG secondary antibody (Invitrogen, Carlsbad, CA, USA; Cat# A-11001; 1:250). Fluorescence images were acquired with a confocal microscope (Leica TCS SP5; 10× objective). Endothelial fibrin deposition was quantified as fibrin-positive area using ImageJ (10 fields/slide).

### Platelet-enriched whole-blood perfusion assay under flow

For platelet preparation and perfusion, 1× Tyrode’s buffer was freshly prepared containing NaCl (8 g/L), NaHCO₃ (1.015 g/L), KCl (0.195 g/L), glucose (1 g/L), and HEPES buffer solution (1% v/v) in water, and adjusted to pH 6.5 or pH 7.2. Citrated blood from healthy donors was diluted 1:1 with Tyrode’s buffer pH 6.5 and centrifuged at 130 × g for 20 min at 20–25 °C without brake. Platelet-rich plasma was collected, diluted in Tyrode’s buffer pH 6.5, supplemented with prostacyclin (100 ng/mL), and centrifuged at 360 × g for 10 min. The platelet pellet was resuspended in Tyrode’s buffer pH 6.5, labelled with Calcein-AM (1 µM, 30 min, 37 °C, Thermo), washed and adjusted to 150,000–200,000 platelets/µL. For flow experiments, µ-slides with flow conditioned endothelial monolayers on both sides were disconnected from the ibidi pump system, washed three times with HBSS, and connected to two 20-mL syringes via a three-way valve. Lithium-heparinized whole blood of the same donor was supplemented with labelled washed platelets (42 × 10^6^ platelets in 4 mL whole blood). Platelet-enriched whole blood was perfused through the slides at 47.4 mL/h, corresponding to approximately 5–8 dyn/cm^2^ depending on the effective blood viscosity. Upon blood perfusion, slides were immediately washed with Tyrode’s buffer supplemented with CaCl₂. Fluorescence images were acquired with a confocal microscope (Leica TCS SP5; 20× objective). Platelet adhesion was quantified as calcein-positive area using ImageJ (10 fields/slide).

### Enzymatic digestion, antioxidant treatment, inflammatory stimulation

To address the contribution of eGCX glycosaminoglycans, HUVEC monolayers were treated for 1 h at 37 °C with heparinase III (1.2 U/mL; Sigma-Aldrich, St. Louis, MO, USA; Cat# H8891) to degrade HS and/or hyaluronidase (4.5 U/mL; Sigma-Aldrich; Cat# H1136) to degrade HA. For antioxidant treatment, HUVECs were incubated with N-acetylcysteine (NAC; 1 mM; Sigma-Aldrich; Cat# A9165), considered as optimal concentration for HUVECs according to Zhang et al. [40], for 16 h before initiation of the IH protocol; NAC was maintained throughout IH exposure. Controls received the respective vehicle. For comparator experiments to investigate inflammation-triggered endothelial SDC-1 release, HUVECs were stimulated with TNF (10 ng/mL; Miltenyi Biotec) for 4 h.

### Immunofluorescence staining of HA, HS and tissue factor (TF)

Following IH, HUVECs were stained for HS using clone F58-10E4 (AMSBIO, Abingdon, United Kingdom; Cat# 370255; 1:100) and detected with a goat anti-mouse IgM Alexa Fluor 488 secondary antibody (Invitrogen; Cat# A-10680; 1:100). HA was stained using biotinylated hyaluronic acid binding protein (b-HABP; AMSBIO/Seikagaku; Cat# 400763) and visualized with Cy3-conjugated streptavidin (BioLegend, San Diego, CA, USA; Cat# 405215); nuclei were counterstained with To-Pro-3 iodide (Thermo Fisher Scientific, Waltham, MA, USA; Cat# T3605). TF was immunostained with anti-CD142 monoclonal antibody (HTF-1; eBioscience™; Catalog # 16–1429-82; 1:100) and detected with a goat anti-mouse IgG Alexa Fluor 488 secondary antibody (Invitrogen; Cat# A- 11001; 1:200). Confocal z-stacks were acquired on a Leica TCS SP5. HA, HS and TF fluorescence was quantified on maximum-intensity projections, using 10 images per slide. Fluorescence intensity was calculated as the background-subtracted mean fluorescence intensity within ROIs covering the endothelial cell monolayer (arbitrary units).

### Flow cytometry and nitric oxide assay

Surface syndecan-1 and TF were stained using FITC anti-human CD138 (clone DL-10; BioLegend; Cat# 352,303; 1:100) and APC anti-human CD142 (BioLegend; Cat# 365205; 1:100) respectively. For intracellular phospho-protein analysis, cells were fixed and permeabilized using the eBioscience™ Intracellular Fixation & Permeabilization Buffer Set and stained with Phospho-AKT1 (Ser473) Monoclonal Antibody (SDRNR), APC, eBioscience™ (1:100) according to the manufacturer’s instructions. As a positive control for phospho-AKT staining, HUVECs were serum-starved for 6 h in basal medium containing 0.5% FCS and then stimulated with VEGF (50 ng/mL, Miltenyi Biotec) for 10 min. Intracellular ROS and reduced thiols were quantified using CellROX Green (Thermo Fisher Scientific; Cat# C10492) and ThiolTracker (Thermo Fisher Scientific; Cat# T10095), respectively, following manufacturers’ instructions; analysis was performed in FlowJo (v10; BD Biosciences). Nitric oxide bioavailability in supernatants was quantified as nitrite using the Measure-IT High-Sensitivity Nitrite Assay Kit (Thermo Fisher Scientific, Waltham, MA, USA; Cat# M36051).

### ELISA

Syndecan-1 was quantified using the Human CD138 (Syndecan-1) ELISA kit (Diaclone, Besançon, France; Cat# 950.640.196). HS was measured using the Heparan sulfate ELISA kit (Wuhan Fine Biotech, Wuhan, China; Cat# ABIN5525972). HA was quantified using the Hyaluronan (HA) ELISA kit (Echelon Biosciences, Salt Lake City, UT, USA; Cat# K-1200), detecting HA species as small as 6.4kDa. Intra- and inter-assay coefficients of variation were, respectively: HS 5.2% and 5.3%; HA < 20% and <10%; SDC-1 6.2% and 10.2%.

Concentrations of secreted MMP-2 and ANGPT-2 were measured using ELISA kits from BioLegend (MMP-2 Cat# 444,607; ANGPT-2 Cat# 440,404). Heparanase (HPSE) was measured using an ELISA kit from antibodies.com (Cat# A78260) and VWF with an ELISA Kit from Invitrogen (Human VWF ELISA Kit, Catalog # EHVWF). Absorbance was measured on a FilterMax F3 plate reader and concentrations were calculated from plate-specific standard curves.

### Quantitative real-time PCR

Total RNA was isolated from HUVECs (miRNeasy Mini Kit, QIAGEN, Hilden, Germany). cDNA was synthesized using oligo-dT primers and random hexamers with Superscript III (Invitrogen, Carlsbad, CA, USA). qRT-PCR was performed on a LightCycler 480 (Roche, Basel, Switzerland) using gene-specific primers/probes (ProbeFinder/Universal ProbeLibrary; Roche, Basel, Switzerland). Primers and Universal ProbeLibrary probe numbers (or assay IDs) are provided in Supplementary Table S1. Reactions were run in duplicate (10 ng cDNA/well). Cq values were determined by the second-derivative maximum method; GAPDH and TBP served as reference genes (PrimePCR Probe Assays; Bio-Rad, Hercules, CA, USA).

### Western blotting

HUVECs were lysed in buffer containing protease and phosphatase inhibitors. Protein concentration was determined by BCA assay. Equal protein amounts were separated by SDS-PAGE and transferred to PVDF membranes (Trans-Blot Turbo, Bio-Rad, Hercules, CA, USA). Membranes were blocked (5% milk/TBST) and incubated with primary antibodies against TRXRD1/TrxR1 (clone B-2; Santa Cruz Biotechnology; Cat# sc-28321; 1:100), eNOS/NOS3 (Santa Cruz Biotechnology; Cat# sc-376751; 1:100), NRF2 (clone A-10; Santa Cruz Biotechnology; Cat# sc-365949; 1:100), and β-actin (Cell Signaling Technology; Cat# 4970; 1:1000). HRP-conjugated secondary antibodies were anti-rabbit IgG (Cell Signaling Technology; Cat# 7074; 1:2000) and anti-mouse IgG (Cell Signaling Technology; Cat# 7076; 1:2000). Bands were visualized with a CCD imager. For Western blot densitometry, paired control/IH experiments were included when target and β-actin bands were clearly detectable, non-saturated, and suitable for reliable normalization. Densitometric target/β-actin ratios were log10-transformed prior to statistical testing to improve distributional symmetry and stabilize variance.

### Plasma proteomics

Plasma samples were randomized on a plate and prepared for LC-MS analysis with the autoSP3 protocol [41] on an Agilent Bravo liquid handling platform (Agilent Technologies, Santa Clara, CA, USA). Briefly, plasma samples were diluted in 100 mM ammonium bicarbonate and 1 µL plasma per sample was used. Reduction and alkylation were performed at 1% SDS, 10 mM TCEP, 40 mM CAA at 95 °C for 10 min. 5 µL of magnetic beads were added (Cytiva, Marlborough, MA, USA; #45152105050250 and #65152105050250 1:1) and proteins bound to beads upon addition of 50% (v/v) acetonitrile (ACN). Beads were washed twice with 80% (v/v) ethanol and once with acetonitrile. Beads were resuspended in 100 mM ammonium bicarbonate and 1.2 µg trypsin were added for overnight digestion at 37 °C. Peptides were acidified to 1% TFA and 0.25 µL of the peptide solution were loaded on Evotip Pure tips (Evosep, Odense, Denmark) with a semi-automated protocol on the Agilent Bravo according to manufacturer’s instructions: tips were washed with ACN/0.1% formic acid (FA), conditioned with 2-propanol, equilibrated with 0.1% FA before sample loading. Tips were washed twice with 0.1% FA and stored in 0.1% FA to prevent drying out.

Evotip Pure tips were placed in the Evosep One (Evosep, Odense, Denmark) autosampler and peptides injected from there using the 30 samples per day method with 44 min gradient. An EV1137 column with C18 material was used (15 cm ×150 µm, 1.5 µm). Eluted peptides were electrosprayed (2kV, 275°C) into an Exploris 480 mass spectrometer (MS) (Thermo Fisher Scientific, Bremen, Germany). The MS was operated in data-independent acquisition (DIA) mode with an MS1-scan at a resolution of 120,000 (at m/z 200), scan range 380–980 m/z, 300% AGC target and 100 ms maximum injection time; followed by 30 MS/MS scans in windows of 20 m/z with 1 m/z overlap and normalized collision energy 30%. MS2 resolution was 30,000 (at m/z 200), AGC target 3000%, auto maximum injection time, and data were recorded in centroid mode.

Proteins were identified and quantified using the software Spectronaut (version 20.1.250624.92449; Biognosys, Schlieren, Switzerland). For identification, directDIA mode and the fasta file “uniprot_sprot_2024-01-01_HUMAN” featuring 20,428 entries downloaded in the software Spectronaut were used. Carbamidomethylation was set as fixed modification, protein N-terminal acetylation and methionine oxidation as variable modifications, and two miscleavages were allowed. Cross-run normalization was activated. Data were exported from Spectronaut. The mass spectrometry proteomics data have been deposited to the ProteomeXchange Consortium via the PRIDE [42] partner repository with the dataset identifier PXD073409.

### Clinical variables, proteomics preprocessing, and statistical analysis

Label-free plasma proteomics was analyzed at the protein-group level. Intensities were log₂-transformed, mapped to representative gene symbols, and proteins with missing values were excluded. Age-adjusted linear models were used for the severe OSA versus non-OSA comparison and for continuous clinical predictors, including AHI, ODI3%, hypoxic burden (HB), nadir O₂, %T90, HA, and HS. Non-OSA was defined as AHI < 5 events/h, mild OSA as AHI 5–15 events/h, and severe OSA as AHI > 30 events/h. *p* values were adjusted using Benjamini–Hochberg FDR, and proteins were retained if quantified in ≥ 10 participants per group.

Differentially expressed proteins were defined by FDR < 0.25 and |log₂FC| ≥0.5 and analyzed by over-representation analysis using MSigDB GO:BP, GO:MF, and KEGG gene sets with clusterProfiler. Preranked GSEA was performed on age-adjusted per-protein statistics for each predictor, and normalized enrichment scores with FDR correction were calculated within each collection. Proteome-wide concordance between OSA-related and HA/HS-related protein signatures was assessed by Spearman correlation of per-protein t-statistic vectors; density-scatter plots included ordinary least-squares regression lines. Proteomic analyses were performed in R v4.5.1 and Python v3.13.4.

Clinical biomarker analyses of circulating HA, HS, and SDC-1 were performed on log10-transformed concentrations using parsimonious multivariable linear models adjusted for age, BMI, hypertension, hs-CRP, fasting glucose, and eGFR. Severity-group analyses used the same covariate set; adjusted geometric means and Tukey-corrected pairwise contrasts were back-transformed from the model scale. Sensitivity analyses additionally included albumin or cholinesterase and were repeated after excluding smokers or participants with hypertension.

Cell-based experiments and biomarker comparisons were analyzed in GraphPad Prism 10.6.1. Normality was assessed by Shapiro–Wilk and D’Agostino–Pearson tests. Paired comparisons used paired t-tests or Wilcoxon signed-rank tests. Comparisons across OSA severity groups or IH paradigms were analyzed by one-way ANOVA with Tukey’s post hoc test or, for non-parametric repeated-measures data, Friedman test with Dunn’s correction. For ordered IH paradigms, linear trends were additionally assessed using GraphPad Prism’s test for linear trend, where appropriate. Data are shown as mean ± SEM unless stated otherwise; N indicates independent experiments (different HUVEC donors).

## Results

While it is well established that OSA promotes various CVD, the relevance of eGCX degradation, an increasingly recognized player in CVD, has only been incompletely defined in this patient cohort. 45 male OSA patients with varying disease severity, classified as mild, moderate, or severe based on the apnea-hypopnea index (AHI) measured during PSG, alongside 15 male age-comparable study participants with PSG or PG based exclusion of OSA (non-OSA, AHI < 5) were examined in this prospective observational study with a cross-sectional clinical biomarker analysis (Table 1: patient characteristics).

**Table 1:** Patient characteristics

Characteristic	Non-OSA (*n* = 15)	Mild OSA (*n* = 15)	Moderate OSA (*n* = 15)	Severe OSA (*n* = 15)	*p*-value (all groups)
Age (years)	35.9 ± 12.3	42.2 ± 7.7	45.1 ± 8.8	43.9 ± 10.4	0.083
BMI (kg/m^2^)	25.6 ± 3.8	27.1 ± 3.3	27.4 ± 2.4	31.5 ± 6.1	<0.001
TST (min)	351.0 ± 54.6	365.2 ± 50.7	321.5 ± 93.7	359.1 ± 68.7	0.359
AHI (events/h)	3.1 ± 1.2	9.8 ± 3.2	20.8 ± 4.6	59.7 ± 17.7	<0.001
ODI 3% (events/h)	1.7 ± 2.2	5.1 ± 3.2	13.5 ± 6.6	33.8 ± 14.2	<0.001
ODI 4% (events/h)	0.9 ± 0.8	2.4 ± 1.9	8.2 ± 5.6	28.8 ± 17.1	<0.001
Max. apnea duration (s)	7.9 ± 12.9	20.8 ± 11.9	31.2 ± 21.3	57.2 ± 59.4	0.006
Mean awake SpO₂ (%)	95.2 ± 0.8	95.1 ± 1.2	95.0 ± 1.2	92.9 ± 2.0	<0.001
Nadir SpO₂ (%)	89.2 ± 2.9	87.2 ± 5.0	82.6 ± 5.7	71.8 ± 13.5	<0.001
%T90 (SpO₂ <90%)	0.0 ± 0.1	1.4 ± 3.2	3.8 ± 3.7	27.8 ± 31.2	<0.001
Hypoxic burden (HB) %min/h	4.7 ± 3.0	19.7 ± 13.9	43.8 ± 19.1	246 ± 216	<0.001
Hypertension, n (%)	2 (13.3%)	0 (0.0%)	2 (13.3%)	6 (40.0%)	0.028
Current smoker, n (%)	1 (6.7%)	0 (0.0%)	0 (0.0%)	1 (6.7%)	0.558
hs-CRP [mg/dL]	0.17 ± 0.08	0.29 ± 0.25	0.18 ± 0.09	0.39 ± 0.41	0.056
Fasting glucose [mg/dL]	90.4 ± 13.4	102.4 ± 21.7	95.2 ± 8.5	100.9 ± 8.4	0.089
eGFR (CKD-EPI) [mL/min/1.73 m^2^]	108.9 ± 11.0	103.5 ± 10.8	96.8 ± 13.8	106.5 ± 12.8	0.050

Values are mean ± SD unless otherwise indicated. Hypertension and current smoking are shown as n (%). *p* values represent overall comparisons across Non-OSA, Mild OSA, Moderate OSA, and Severe OSA. In the non-OSA group, TST, mean awake SpO₂, and HB were available only for the polysomnography subset (n = 5). Known diabetes was an exclusion criterion. Abbreviations: OSA, obstructive sleep apnea; BMI, body mass index; TST, total sleep time; AHI, apnea-hypopnea index; ODI, oxygen desaturation index; SpO₂, peripheral oxygen saturation; T90, percentage of TST with SpO₂ <90%; HB, hypoxic burden; hs-CRP, high-sensitivity C-reactive protein; eGFR, estimated glomerular filtration rate

### OSA patients exhibit significantly elevated plasma levels of eGCX glycosaminoglycans hyaluronan and heparan sulfate

**Fig. 1:**
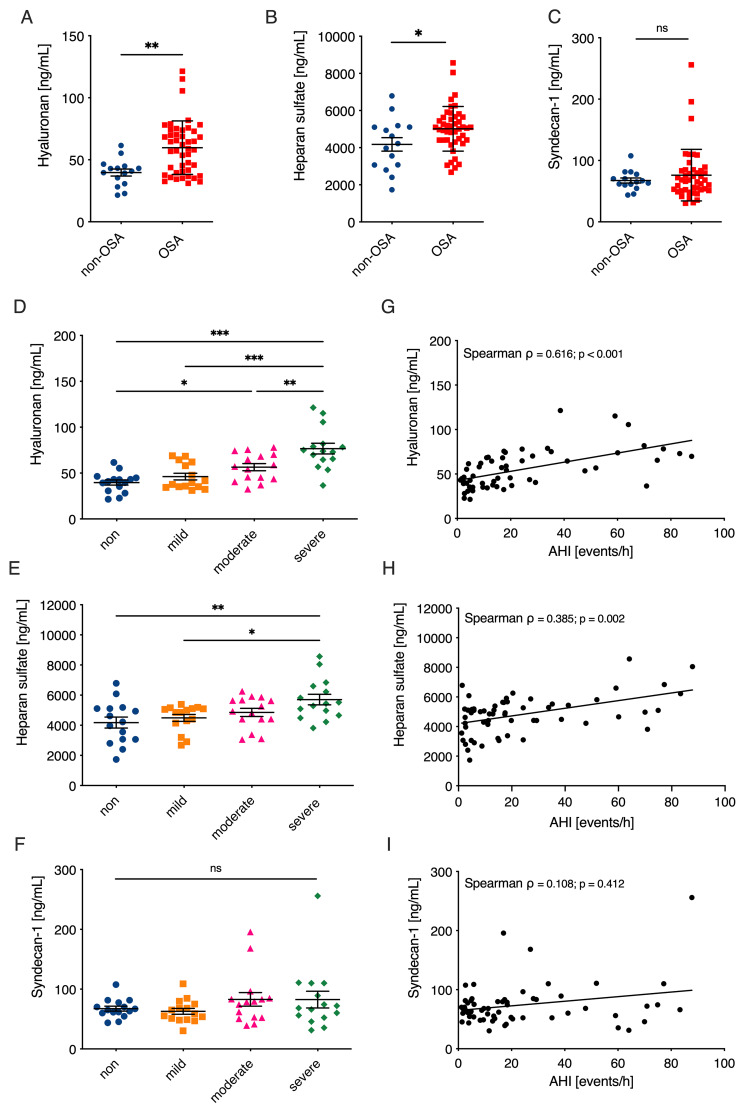
Plasma concentrations of essential eGCX components HA, HS, and SDC-1 in OSA patients as compared to non-OSA controls. (**A-C**) eGCX plasma concentrations of HA, HS, and SDC-1 as assessed by ELISA for age-comparable non-OSA controls (*n* = 15) and OSA-patients of different disease severity (*n* = 45, mean±SD). (**D**, **E**, **F**) eGCX plasma concentrations of HA, HS, and SDC-1 determined by ELISA analyses in non-OSA controls and OSA-patients grouped by AHI-based OSA severity (mild: AHI 5–15, *n* = 15; moderate: AHI 15–30, *n* = 15; severe: AHI > 30, *n* = 15) (mean±SEM for *n* = 15 per group; *p < 0.05, **p < 0.01, and ***p < 0.001 vs non-OSA, mild, moderate, or severe, tukey-adjusted *p*-values). (**G**, **H**, **I**) statistical correlation analyses between plasma levels of eGCX components HA, HS, and SDC-1 and AHI as a measure of disease severity

OSA patients exhibited significantly increased plasma levels of key eGCX glycosaminoglycans hyaluronan (Fig. 1A, HA: non-OSA mean ± SD 39.71 ± 10.96 ng/mL vs. OSA patients 59.75 ± 21.55 ng/mL, *n* = 15 vs. 45, *p* < 0.001) and heparan sulfate (Fig. 1B, HS: non-OSA mean ± SD 4176.93 ± 1412.07 ng/mL vs. OSA patients 5015.83 ± 1201.32 ng/mL, *n* = 15 vs. 45, *p* = 0.029) when compared to non-OSA controls. In contrast, plasma levels of the proteoglycan SDC-1 (Fig. 1C, SDC-1: non-OSA, 67.40 ± 15.76 ng/mL vs. OSA patients 76.10 ± 42.01 ng/mL, *n* = 15 vs. 45, *p* = 0.826), one of the most relevant eGCX backbone molecules, did not significantly differ between OSA patients and non-OSA controls.

Increasing disease severity was associated with higher mean plasma concentrations of HA and HS, and HA plasma levels differed significantly between all three disease severity groups and the non-OSA group (Fig. 1D, HA: non-OSA 39.71 ± 10.96 ng/mL, mild 46.14 ± 14.11 ng/mL, moderate 56.45 ± 15.04 ng/mL, severe 76.67 ± 22.73 ng/mL; Fig. 1E, HS: non-OSA 4176.93 ± 1412.07 ng/mL, mild 4490.58 ± 884.71 ng/mL, moderate 4850.87 ± 1044.38 ng/mL, severe 5706.04 ± 1351.70 ng/mL; *n* = 15 per group, Fig. 1F, SDC-1: non-OSA 67.40 ± 15.76 ng/mL, mild 61.68 ± 19.10 ng/mL, moderate 82.87 ± 44.11 ng/mL, severe 82.55 ± 54.36 ng/mL; *n* = 15 per group). Notably, plasma HA and HS concentrations positively correlated with AHI (Fig. 1G, HA: Spearman’s ρ=0.616, *p* < 0.001; Fig. 1H, HS: ρ=0.385, *p* = 0.002), whereas SDC-1 showed no association (Fig. 1I, ρ=0.108, *p* = 0.412). HA and HS were also associated with oximetry-derived hypoxic exposure, including ODI3% (HA: ρ=0.539, *p* < 0.001; HS: ρ=0.362, *p* = 0.005), hypoxic burden (HB; HA: ρ=0.523, *p* < 0.001; HS: ρ=0.358, *p* = 0.01), while SDC-1 was not (Supplementary Table S2).

In additional analyses, we tested whether associations of circulating glycocalyx-related markers with OSA severity and hypoxemia were robust to broader adjustment. In parsimonious multivariable models for log10-transformed HA, HS, and SDC-1 including age, BMI, hypertension, hs-CRP, fasting glucose, and eGFR, higher AHI and ODI3% both remained associated with higher HA and HS, whereas SDC-1 did not show consistent associations. Results for HB were directionally similar but less precise in the smaller complete-case subset. In adjusted severity-group analyses based on the same covariate set, HA remained higher across OSA strata, HS showed a slightly weaker but concordant pattern, and SDC-1 data remained comparable. Sensitivity analyses including albumin or cholinesterase, excluding smokers, and excluding participants with hypertension did not materially alter the overall pattern. Adjusted geometric means across the four OSA severity strata are shown in Supplementary Fig. S2, and full multivariable and sensitivity analyses are provided in Supplementary Tables S3–S5.

### An OSA-related model of rapid-cycling IH under flow promotes degradation of glycocalyx glycosaminoglycans HS and HA, but not of SDC-1

**Fig. 2:**
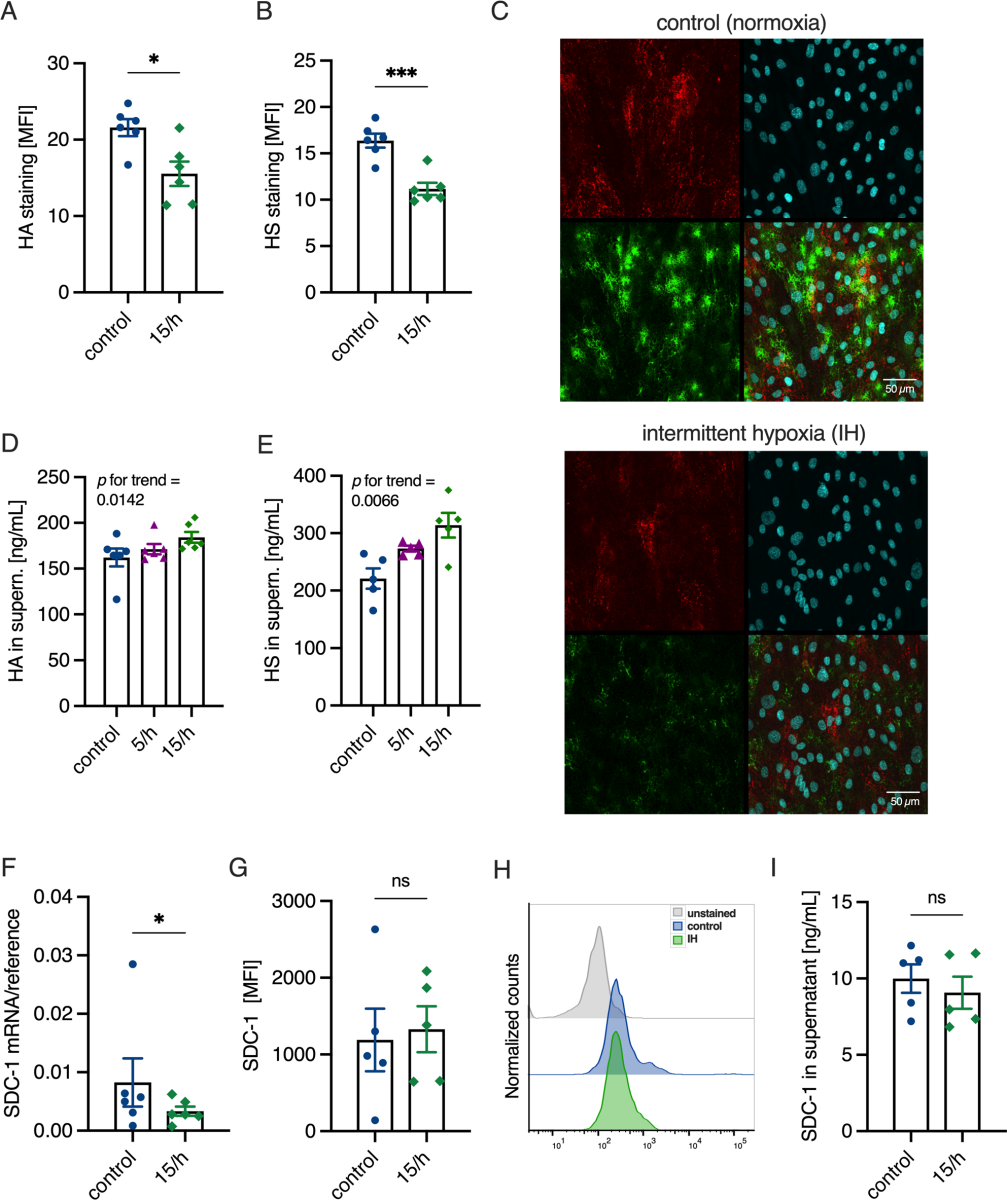
eGCX integrity in the OSA-characteristic in vitro intermittent hypoxia (IH) model. (**A, B**) intensity of HA and HS immunostaining on endothelial shear-matured monolayers subjected to flow (15 dyn/cm^2^) for 5 days and subsequently to IH (15 hypoxic cycles per hour for 4 hours; higher-burden IH). (**C**) representative images of HA (red), HS (green), and nuclei (blue) immunostaining after IH. Scale bar: 50 µm. (**D, E**) concentrations of shed HA and HS in cell-culture supernatant as assessed by ELISA after normoxic control conditions or graded IH exposure at 5 and 15 cycles/h. (**F**) endothelial SDC-1 mRNA expression measured by qPCR and (**G**) extracellular SDC-1 protein expression on endothelial cells measured by flow cytometry (MFI) after IH as compared to control. (**H**) representative flow-cytometry histogram (overlay) of SDC-1 fluorescence (MFI) in unstained, control, and IH-treated endothelial cells. (**I**) shed SDC-1 protein concentrations in cell-culture supernatant. IH, intermittent hypoxia. Data are mean ± SEM for *n* = 5–6 independent experiments; *p < 0.05, ***p < 0.001; ns, not significant

To explore whether the increase in circulating HA and HS observed in patients with OSA could be recapitulated in an endothelial model, we used a previously established reductionistic in vitro system of OSA-like IH under arterial flow in shear stress-matured human endothelial cells [19]. In the higher-burden condition (15 cycles/h for 4 h), IH reduced surface staining of HA (Fig. 2A, −28.02%±6.87, *n* = 6, *p* = 0.016) and HS (Fig. 2B, −31.76%±3.40, *n* = 6, *p* < 0.001; Fig. 2C, representative confocal images). We then compared previously validated lower- and higher-burden IH paradigms (5/h and 15/h for 4 h) [19]. In these experiments, supernatant HA increased from 162.19 ± 9.84 ng/mL in control to 171.27 ± 5.61 ng/mL at 5/h and 184.20 ± 5.86 ng/mL at 15/h (Fig. 2D, *n* = 6 per condition), consistent with a significant linear increase across increasing IH burden (p for trend = 0.0142). Supernatant HS likewise increased from 220.98 ± 17.80 ng/mL in control to 273.28 ± 4.74 ng/mL at 5/h and 313.84 ± 21.53 ng/mL at 15/h (Fig. 2E, *n* = 5 per condition), also showing a significant linear increase across increasing IH burden (p for trend = 0.0066). While SDC-1 mRNA decreased after IH (Fig. 2F, control vs. 15/h −59.72%±11.03, *n* = 6, *p* = 0.031), surface SDC-1 (Fig. 2G/H, control 1189.6 ± 407.2 vs. IH 1328.0 ± 299.4, +11.63%±173.49, *n* = 5, *p* = 0.827) and soluble SDC-1 (Fig. 2I, control 10.00 ± 0.94 ng/mL vs. IH 9.07 ± 1.05 ng/mL, −9.26%±6.34, *n* = 5, *p* = 0.262) remained unchanged. Thus, OSA-like IH promoted HA/HS-related glycocalyx remodeling, whereas overt SDC-1 shedding was not observed.

To further address this dissociation, we performed comparator experiments with TNF as an established, strong inflammatory stimulus with high potential for eGCX shedding. In contrast to IH, TNF significantly increased soluble SDC-1 in the supernatant, reduced surface SDC-1, induced a more pronounced decrease in SDC-1 mRNA, and markedly enhanced MMP-2 release indicating that the used assay system can detect canonical SDC-1 shedding and supporting a stimulus-specific pattern of glycocalyx remodeling (Supplementary Fig. S3).

### Plasma proteome analysis identifies oxidative-stress and procoagulant-inflammatory signatures associated with OSA burden

**Fig. 3:**
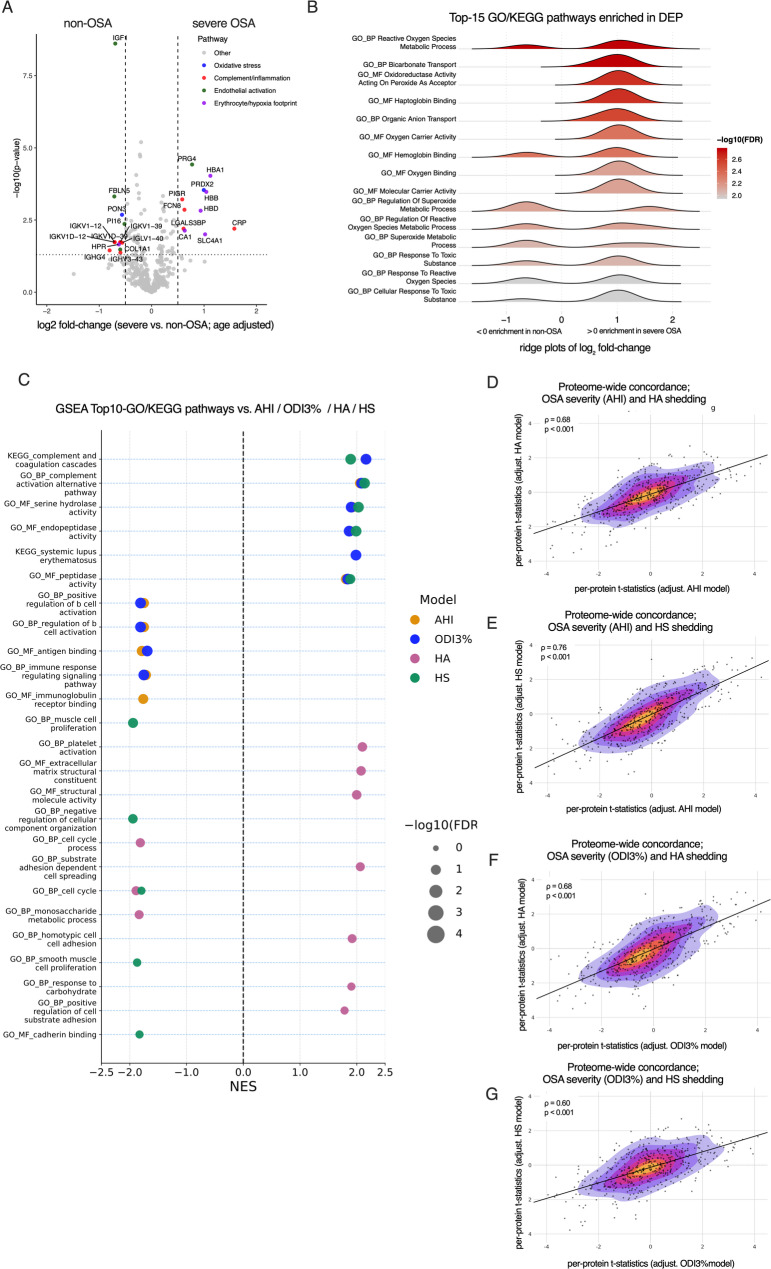
Plasma proteome analysis. (**A**) volcano plot of age-adjusted plasma protein abundance comparing non-OSA controls with severe OSA patients (contrast: severe vs non-OSA). Selected proteins and their associated pathways (colored) are highlighted (−log10 p > 1.3 and |log2FC| >0.5). (**B**) Over-representation analysis of Gene Ontology biological process (GO-BP), molecular function (GO-MF) and KEGG pathways based on differentially expressed proteins (DEP) from the age-adjusted comparison between severe OSA and non-OSA. Shown are the top-15 enriched pathways (ranked by FDR) as ridgeline density plots of DEP log2 fold-changes (severe vs non-OSA); curves shifted to the right indicate pathways predominantly upregulated in severe OSA, whereas curves shifted to the left indicate higher abundance in non-OSA. (**C**) preranked gene set enrichment analysis (GSEA) of plasma protein abundance adjusted for age modeled as a function of OSA severity or hypoxemia burden (AHI, ODI3%) and circulating glycocalyx-related markers HA and HS individually. Shown are top 10 enriched GO/KEGG pathways with normalized enrichment scores (NES); dot size indicates −log10(FDR). Positive NES indicates enrichment with higher predictor values. (**D–G**) proteome-wide concordance analyses comparing per-protein t-statistics from age-adjusted models for OSA burden and circulating HA/HS: AHI versus HA (**D**), AHI versus HS (**E**), ODI3% versus HA (**F**), and ODI3% versus HS (**G**). Spearman correlation coefficients and p values are shown in each panel

To explore molecular pathways that link OSA to eGCX degradation and vascular dysfunction, we performed an unbiased plasma proteomics analysis in a subset of the cohort (15 non-OSA controls, 15 patients with mild OSA, and 15 patients with severe OSA). Using an age-adjusted linear model comparing severe OSA with non-OSA controls (FDR < 0.25, |log_2_FC|>0.50), we identified 11 proteins that were upregulated in the severe group and 13 proteins that were relatively higher in non-OSA controls (Fig. 3A, volcano plot; Supplemental Table S6). Among the proteins increased in severe OSA, the strongest signal was the inflammation-associated acute-phase reactant C-reactive protein (CRP; log_2_FC 1.59, q = 0.071), together with oxidative-stress–related proteins such as peroxiredoxin-2 (PRDX2; log_2_FC 1.15, q = 0.0097). The most significantly downregulated protein in this group was insulin-like growth factor-1 (IGF-1; log_2_FC −0.699, q < 0.001), consistent with a relative loss of IGF1-mediated vascular protection in severe OSA. Similar directional trends were seen in the group comparisons of non-OSA vs mild and mild vs severe, showing higher CRP and PRDX2 (and lower IGF-1 level) in the group with higher OSA burden, although these differences did not meet the predefined FDR threshold (Supplemental Table S7, S8).

Over-representation analysis using these differentially regulated proteins in clusterProfiler highlighted GO biological process and molecular function terms, as well as KEGG pathways, related to antioxidant activity and cellular stress responses (Fig. 3B, ridgeline plot of top 15 ranked by FDR, Supplemental Table S9), supporting a systemic oxidative stress signature in patients with severe OSA.

We next modelled protein abundance as a function of AHI, ODI3% and circulating HA and HS individually, with adjustment for age, to obtain signals related to OSA event burden (AHI), hypoxemia burden (ODI3%), and circulating glycocalyx-related markers. Gene set enrichment analysis using preranked lists for each predictor revealed overlapping positive enrichment of complement/coagulation and protease-related pathways for AHI, ODI3%, and HS, whereas HA-associated signatures were dominated by platelet activation, extracellular matrix organization, and cell-adhesion-related pathways (Fig. 3C, dotplot with normalized enrichment scores, Supplementary Table S10).

Importantly, proteome-wide t-statistics from either the AHI model (HA: Fig. 3D, ρ=0.68; HS: Fig. 3E, ρ=0.76) or the ODI3% model (HA: Fig. 3F, ρ=0.68; HS: Fig. 3G, ρ=0.60) were correlated with the corresponding models using HA and HS, indicating that proteins tracking OSA severity also tracked circulating glycocalyx-related markers (Supplemental Table S11).

Taken together, these unbiased human plasma proteomics data support a model in which IH-driven inflammatory and oxidative stress responses promote eGCX degradation and a procoagulant, inflammatory vascular state.

### Molecular mechanisms of eGCX degradation and endothelial dysfunction

**Fig. 4:**
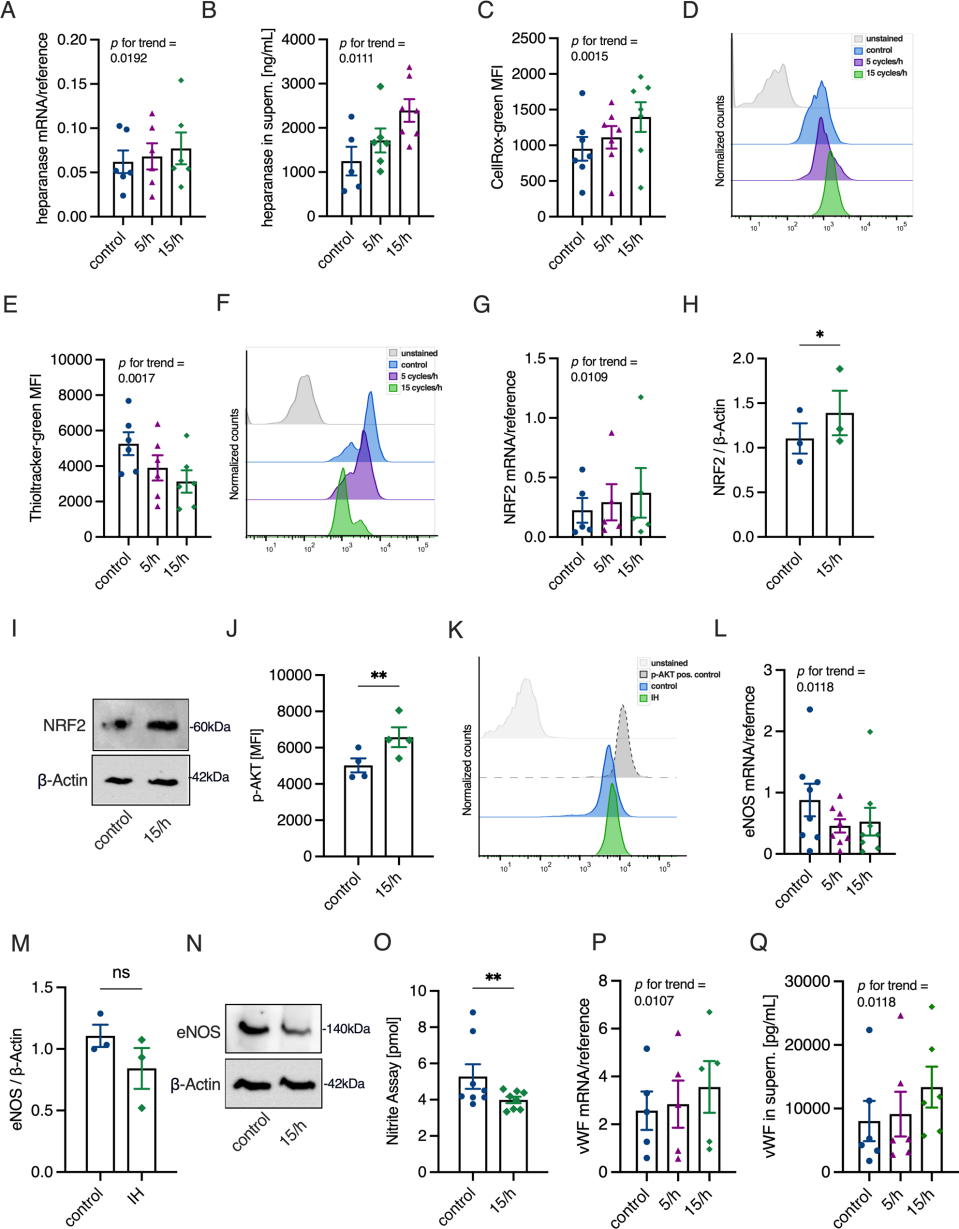
Molecular mechanisms assessed in the OSA-characteristic in vitro IH model. (**A, B**) endothelial heparanase (HPSE) mRNA expression measured by qPCR and HPSE protein concentrations in cell-culture supernatants measured by ELISA after normoxic control conditions or graded IH exposure at 5 and 15 cycles/h. (**C**) intracellular reactive oxygen species quantified by CellROX green fluorescence using flow cytometry. (**D**) representative flow-cytometry histogram of CellROX fluorescence in unstained, control, 5 cycles/h, and 15 cycles/h conditions. (**E**) intracellular reduced thiols quantified by ThiolTracker green fluorescence as a readout of cellular redox capacity. (**F**) representative flow-cytometry histogram of ThiolTracker fluorescence. (**G–I**) NRF2 expression assessed by qPCR (**G**), Western blot densitometry normalized to β-actin (**H**), and representative Western blot images (**I**). (**J, K**) intracellular phospho-AKT Ser473 quantified by flow cytometry (**J**) with representative histogram including unstained and positive-control samples (**K**). (**L–N**) endothelial nitric oxide synthase (NOS3/eNOS) expression assessed by qPCR (**L**), β-actin-normalized Western blot densitometry (**M**), and representative Western blot images (**N**). (**O**) nitric oxide bioavailability assessed by nitrite concentrations in cell-culture supernatants. (**P, Q**) von Willebrand factor (vWF) mRNA expression measured by qPCR (**P**) and vWF protein concentrations in cell-culture supernatants measured by ELISA (**Q**). Data are mean ± SEM; *n* = 3–8 independent experiments, depending on assay; *p < 0.05, **p < 0.01; ns, not significant

To elucidate the potential endothelial mediators of eGCX degradation suggested by the plasma proteome, we examined graded responses to lower- and higher-burden IH in our rapid-cycling endothelial model under flow (5 cycles/h and 15 cycles/h for 4 h, respectively). Across these conditions, expression of one of the key eGCX sheddases heparanase (HPSE) increased at both the mRNA level (Fig. 4A, control 0.062 ± 0.013, 5/h 0.068 ± 0.015, 15/h 0.077 ± 0.018, *n* = 6, significant linear trend across conditions, *p* = 0.019) and the protein level in the cell-culture supernatant (Fig. 4B, control 1246.99 ± 295.60 pg/mL, 5/h 1717.12 ± 271.52 pg/mL, 15/h 2394.28 ± 260.59 pg/mL, *n* = 5–7, significant linear trend, *p* = 0.011). Additional glycocalyx-remodeling mediators showed concordant changes in the higher-burden IH condition, including increased MMP2 at the mRNA and supernatant protein level and increased ANGPT2 at the mRNA and protein level (Supplementary Fig. S3). HYAL1 was unchanged, and HYAL2 transcripts were not reliably detectable under the present conditions (Supplementary Fig. S3).

As HA can additionally be shed via oxidative stress-related mechanisms [43], we next assessed endothelial redox stress. IH induced a burden-dependent increase in intracellular ROS, quantified by CellROX (Fig. 4C, control 951.2 ± 166.0, 5/h 1112.6 ± 157.0, 15/h 1396.2 ± 208.5, *n* = 7, significant linear trend, *p* = 0.0015; Fig. 4D, representative histogram), and a reciprocal decrease in intracellular reduced thiols, quantified by ThiolTracker (Fig. 4E, control 5262.2 ± 643.5, 5/h 3899.7 ± 713.5, 15/h 3130.5 ± 632.3, *n* = 6, significant linear trend, *p* = 0.0017; Fig. 4F, representative histogram), indicating progressive oxidative stress and impaired redox capacity. Consistent with this pattern, NRF2 expression was increased at the mRNA level in the higher-burden IH condition, and this was confirmed by Western blot densitometry (Fig. 4G, qPCR: +36.42%±13.51, *n* = 6, significant linear trend, *p* = 0.0109; Fig. 4H WB: control vs. IH +25.80%±5.18, *n* = 3, *p* = 0.034; Fig. 4I, representative blot). TRXRD1 was also upregulated in the higher-burden IH condition (Supplementary Fig. S3).

To further characterize endothelial dysfunction, we assessed Akt signaling, eNOS expression, and NO bioavailability. Endothelial phospho-AKT (Ser473) increased under IH (Fig. 4J, control 5021.5 ± 384.9 vs. IH 6577.0 ± 547.7, +31.14%±4.32, *n* = 4, *p* = 0.0083, Fig. 4K, representative histogram). In contrast, endothelial NOS3/eNOS expression was reduced under IH, with lower NOS3 mRNA levels in the higher-burden IH condition compared with control (Fig. 4L, control 0.880 ± 0.265, 5/h 0.458 ± 0.109, 15/h 0.527 ± 0.227, −40.12%±12.15 at 15/h vs. control, *n* = 8, *p* = 0.037). Western blot densitometry showed a concordant but non-significant reduction in eNOS protein expression after 15/h IH (Fig. 4M, N). In parallel, nitrite levels in the supernatant were reduced after IH (Fig. 4O, control 5.27 ± 0.68 vs. 15/h 3.99 ± 0.18, −18.97%±7.10, *n* = 8, *p* = 0.0078), consistent with impaired endothelial NO bioavailability. In addition, von Willebrand factor (vWF) expression increased across control, 5/h, and 15/h conditions at both the mRNA level (Fig. 4P, control 2.57 ± 0.80, 5/h 2.84 ± 0.99, 15/h 3.56 ± 1.08, +38.60%±12.54 at 15/h vs. control, *n* = 5, significant linear trend, *p* = 0.0107) and the protein levels in the cell-culture supernatant (Fig. 4Q, control 8036.7 ± 3156.4 pg/mL, 5/h 9124.7 ± 3526.3 pg/mL, 15/h 13,375.7 ± 3217.1 pg/mL, +66.43%±38.44 at 15/h vs. control, *n* = 6, significant linear trend, *p* = 0.0118), in line with previous work linking vWF to hypoxia-associated endothelial dysfunction [44].

Together, these findings indicate that OSA-like IH induces a broader endothelial stress response characterized by HPSE-associated glycocalyx remodeling, oxidative stress, altered Akt signaling, endothelial activation, and impaired NO signaling.

### IH-induced eGCX degradation promotes monocyte and platelet adhesion, tissue factor surface expression, and fibrin deposition on flow-cultured endothelial cells

**Fig. 5:**
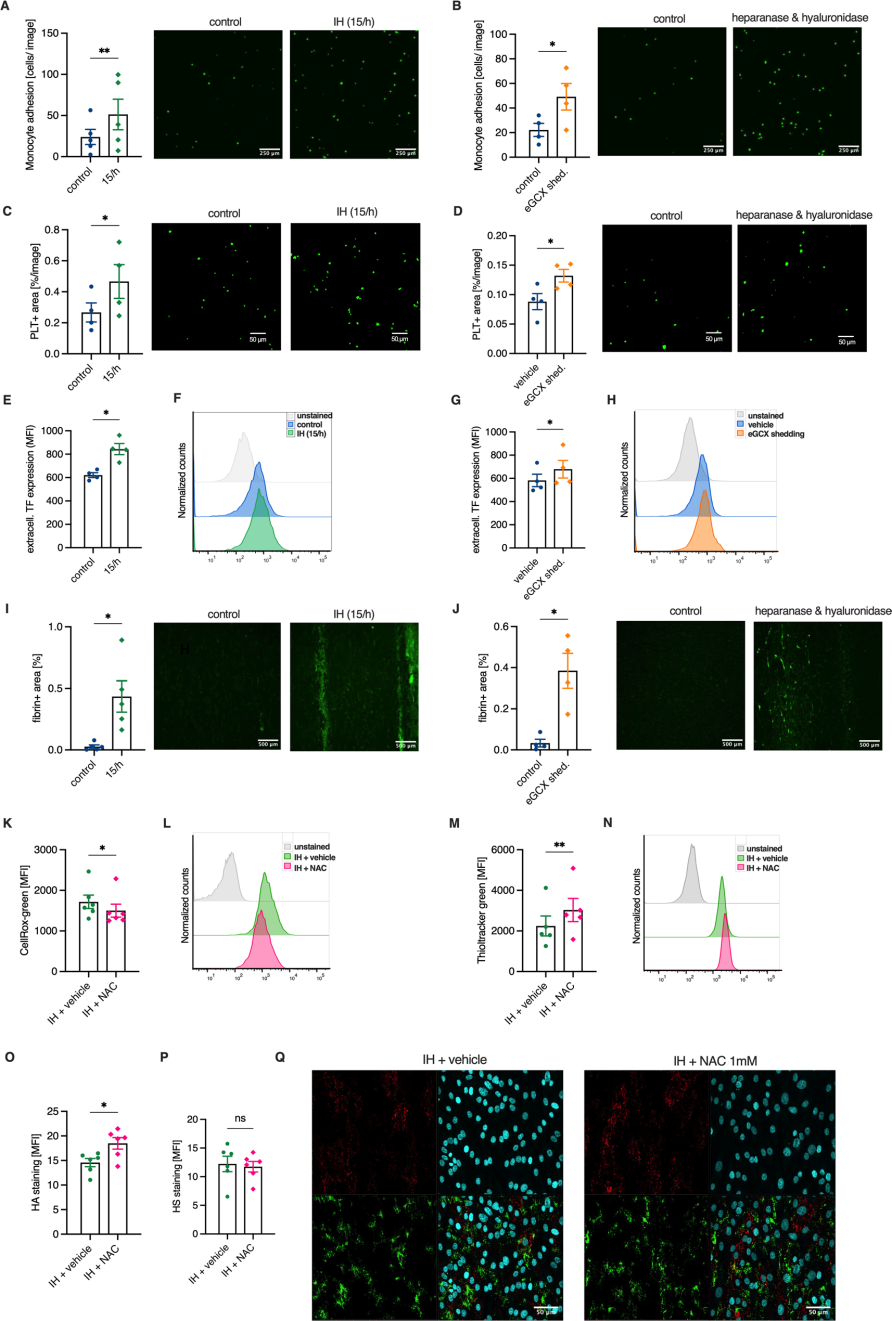
Intermittent hypoxia induces a proadhesive and prothrombotic endothelial phenotype and antioxidant intervention partially limits oxidative stress-associated eGCX perturbation. (**A, B**) monocyte adhesion to shear-matured endothelial monolayers under flow after OSA-mimicking IH at 15 cycles/h (**A**) or after enzymatic eGCX degradation by heparinase III and hyaluronidase (**B**), with representative fluorescence images. Scale bars: 250 µm. (**C, D**) platelet adhesion quantified as calcein-positive platelet area after IH (**C**) or enzymatic eGCX degradation (**D**), with representative images. Scale bars: 50 µm. (**E, F**) endothelial surface tissue factor (TF/CD142) after IH, quantified by flow cytometry (**E**), with representative histogram (**F**). (**G, H**) endothelial surface TF/CD142 after enzymatic eGCX degradation, quantified by flow cytometry (**G**), with representative histogram (**H**). (**I, J**) fibrin deposition after IH (**I**) or enzymatic eGCX degradation (**J**), quantified as fibrin-positive area after whole-blood perfusion, with representative images. Scale bars: 500 µm. (**K, L**) effect of NAC (1 mM, 16 h) on IH-induced intracellular ROS measured by CellROX green fluorescence (**K**), with representative histogram (**L**). (**M, N**) effect of NAC on intracellular reduced thiols measured by ThiolTracker green fluorescence (**M**), with representative histogram (**N**). (**O–Q**) surface HA (**O**) and HS (**P**) fluorescence intensity after IH with NAC pretreatment compared with vehicle, with representative confocal images of HA (red), HS (green), and nuclei (blue) (**Q**). Scale bars: 50 µm. Data are mean ± SEM; *n* = 4–6 independent experiments, depending on assay; *p < 0.05, **p < 0.01; ns, not significant

Since the plasma proteome analysis pointed towards a procoagulant and proinflammatory endothelial state, we next assessed the functional consequences of IH-induced eGCX degradation on intravascular inflammation and thrombogenicity. Both IH exposure and enzymatic degradation of HA and HS increased monocyte firm adhesion to the endothelial monolayer (Fig. 5A, IH: +114.20%±39.11, *n* = 5, *p* = 0.0085; Fig. 5B, enzymes: +121.51%±58.14, *n* = 4, *p* = 0.0482). Platelet adhesion was likewise increased after both IH exposure and enzymatic glycocalyx degradation (Fig. 5C, control 0.267 ± 0.061 vs. IH 0.466 ± 0.109, +74.82%±21.35, *n* = 4, *p* = 0.024; Fig. 5D, control 0.088 ± 0.014 vs enzymes 0.132 ± 0.011, +49.90%±19.86, *n* = 4, *p* = 0.036). Surface tissue factor expression was also increased after both IH exposure (Fig. 5E, control 620.75 ± 20.87 vs. IH 843.50 ± 47.80, +35.88%±9.63, *n* = 4, *p* = 0.031; Fig. 5F, representative histogram), and enzymatic glycocalyx degradation (Fig. 5G, control 582.25 ± 53.45 vs. enzymes 679.00 ± 76.07, +16.62%±3.10, *n* = 4, *p* = 0.012; Fig. 5H, representative histogram). This was supported by significantly increased F3 mRNA and by significantly higher endothelial surface TF expression on immunofluorescence after IH (Supplementary Fig. S4). Consistent with a prothrombotic endothelial shift, fibrin deposition was markedly enhanced after both IH and enzymatic glycocalyx degradation (Fig. 5I, control 0.027 ± 0.014 vs. IH 0.435 ± 0.127, *n* = 5, *p* = 0.036; Fig. 5J, control 0.033 ± 0.019 vs enzymes 0.385 ± 0.085, *n* = 4, *p* = 0.041). Effective degradation of surface-bound eGCX components by enzymatic treatment was confirmed by reduced HA and HS staining (Supplemental Fig. S4).

In a translational follow-up approach, we investigated the eGCX-protective potential of N-acetylcysteine (NAC) in experimental IH. HUVECs pretreated under flow with 1 mM NAC for 16 h before IH exposure showed reduced ROS levels together with increased intracellular thiol groups (Fig. 5K, CellROX: −12.65%±4.11, *n* = 6, *p* = 0.0312; Fig. 5L, representative histogram; Fig. 5M, ThiolTracker: +34.97%±5.19, *n* = 5, *p* = 0.0026; Fig. 5N, representative histogram). While NAC did not restore HS surface levels, it significantly increased HA expression on the endothelial surface after IH (Fig. 5O, HA: +26.89%±11.70, *n* = 6, *p* = 0.0274; Fig. 5P, HS: −3.98%±6.43, *n* = 6, *p* = 0.540; Fig. 5Q, representative confocal images of HA and HS staining). These findings indicate that redox modulation can partially attenuate IH-associated glycocalyx perturbation at least in vitro, supporting antioxidant/eGCX-stabilizing strategies as candidates for future study.

## Discussion

This study integrates clinical biomarkers, unbiased plasma proteomics, and a shear-matured endothelial IH model to examine whether OSA is associated with circulating markers consistent with endothelial glycocalyx perturbation. In a prospectively recruited cohort with a cross-sectional clinical analysis, plasma levels of HA and HS were higher in OSA patients, increased stepwise with AHI severity, and remained associated with hypoxemia indices after multivariable and sensitivity analyses, whereas plasma SDC-1 did not differ between groups. In the endothelial IH model, OSA-like IH reduced surface HA/HS, increased their shedding, induced oxidative stress, impaired NO signaling, and promoted a proadhesive and thrombogenic endothelial phenotype under flow conditions. Together, these findings provide complementary clinical and experimental evidence for OSA-related glycocalyx perturbation and endothelial dysfunction. While the human cohort supports an association between OSA burden and circulating markers consistent with glycocalyx perturbation, mechanistic interpretation derives primarily from the endothelial IH model.

OSA-related vascular injury reflects repetitive oxygen desaturation, together with additional pathophysiological stressors, including sympathetic surges, negative intrathoracic pressure swings, and intermittent hypercapnia which converge on oxidative stress, inflammation, and disturbed shear sensing [16]. CPAP improves symptoms and several vascular surrogates, yet did not reduce major cardiovascular events in a large randomized trial, underscoring the need for targeted adjunctive strategies [8]. In this context, the glycocalyx represents a plausible vascular interface through which hypoxemia-related endothelial stress may be translated into downstream vascular dysfunction. Composite measures of nocturnal desaturation burden are increasingly linked to cardiovascular risk and of potential treatment benefit [4, 5, 9]. In our data, proteomic alterations intensified with higher AHI, and HA/HS plasma levels tracked both AHI and ODI, consistent with a burden-dependent endothelial surface perturbation rather than a simple binary OSA/non-OSA effect.

As circulating HA and HS may be influenced by obesity, inflammation, metabolic dysfunction, and renal or hepatic function, we expanded the clinical biomarker analyses beyond age/BMI-adjusted models. The persistence of the associations of HA and HS with AHI and ODI3% after broader adjustment, together with concordant sensitivity analyses indicates that these associations are not solely triggered by general cardiometabolic burden, although a residual confounding effect cannot be excluded.

Prior OSA studies have mainly relied on surrogate markers such as heparanase and hyaluronidase-1, or the endothelial proteoglycan endocan/ESM-1 [11, 27, 28, 30, 45], whereas direct measurements of core glycocalyx constituents remain scarce and partly inconsistent. Altered circulating HA in OSA has been reported previously [26, 32], so the present HA finding should be interpreted as confirmatory, whereas systematic data on circulating HS across the OSA spectrum are sparse. By quantifying both HA and HS across the OSA spectrum and pairing these readouts with an IH model calibrated to patient-level hypoxic event loads, we provide integrated clinical and mechanistic support that higher circulating HA and HS levels are linked to OSA severity in patients, while OSA-like IH can induce eGCX remodeling in vitro.

The absence of intergroup differences in circulating SDC-1, despite robust HA/HS signals, is noteworthy. Syndecan ectodomain shedding is highly context- and time-dependent and is influenced by MMP/ADAM activity, heparanase processing, clearance kinetics, and vascular-bed-specific release [46–50]. In our IH model, SDC-1 mRNA decreased, whereas surface and soluble SDC-1 remained unchanged. Together with the TNF comparator experiments, our data support stimulus-specific glycocalyx remodeling: Under OSA-like IH, HA/HS-related remodeling appears more prominent without overt syndecan-1 shedding, despite increased MMP-2. In contrast, TNF induced a broad glycocalyx shedding pattern with increased soluble SDC-1 and MMP-2, reduced surface SDC-1, and a stronger decrease in SDC-1 mRNA. Consistent with this interpretation, marked elevations of circulating SDC-1 are most commonly reported in settings of major tissue injury and severe systemic inflammation, such as trauma and sepsis [51, 52], whereas more moderate systemic stress may be accompanied by only limited or inconsistent SDC-1 degradation [53, 54]. Thus, HA and HS might be more sensitive markers of low-grade endothelial surface perturbation in OSA, whereas SDC-1 may reflect a marker for stronger, inflammation-associated proteoglycan remodeling.

In the endothelial IH model, HPSE increased across lower- and higher-burden IH conditions whereas HYAL1 remained unchanged and HYAL2 transcripts were not reliably detectable. These results support preferential activation of a heparanase-associated shedding axis, although direct enzymatic activity measurements will be required to define the responsible shedding pathways more precisely. IH also induced a graded increase in ROS together with reduced cellular thiols and upregulation of NRF2 and TRXRD1, consistent with oxidative stress and compensatory redox responses. Endothelial phospho-AKT increased under IH, whereas NOS3 mRNA expression decreased, eNOS protein abundance showed a concordant reduction, and nitrite levels were reduced, indicating altered upstream signaling that was insufficient to preserve endothelial NO bioavailability. The increase in vWF across control, 5/h, and 15/h conditions further supports endothelial activation under increasing IH burden.

Differential protein abundance and pathway enrichment implicate complement activation, coagulation/fibrinolysis activation, and oxidative stress-related inflammatory programs. These signals intensified with worsening AHI and ODI3% and tracked circulating HA and HS individually. Our functional assays provide a direct link from mechanism to phenotype: IH or enzymatic digestion of HA/HS increased monocyte adhesion, platelet adhesion, tissue factor surface expression, and fibrin deposition on the endothelial monolayer under flow. Consistent with the increased monocyte adhesion observed here, our previously published work using the same shear-matured rapid-cycling IH model showed a graded inflammatory endothelial response across control, 5/h, and 15/h conditions, including endothelial ICAM-1 and CCL2 expression, together with progressively enhanced ERK- and NF-κB-pathway activation [19]. Glycocalyx shedding has been linked to significant adverse cardiovascular and cerebrovascular events [55, 56], supporting the clinical plausibility of this thrombo-inflammatory framework.

Two translational implications emerge from these data. First, HA and HS were linked to both OSA severity and nocturnal desaturation and mirrored a proteomic signature of complement/coagulation and oxidative stress [4, 5, 9]. This suggests that measuring plasma HA and HS levels might help to identify patients with OSA who show biomarker patterns consistent with glycocalyx perturbation, and subsequently, a potentially higher cardiovascular risk. However, the present study was not designed to establish predictive thresholds, quantify incremental value beyond established OSA severity indices, or support clinical risk stratification: These applications will require dedicated longitudinal studies with predictive modeling and external validation. Second, our in vitro data indicate that N-acetylcysteine attenuates ROS generation and partially restores surface HA after IH, consistent with redox-sensitive regulation of eGCX integrity [38]. Preservation of HS, by contrast, may require additional strategies, such as dampening heparanase activity or counteracting angiopoietin-2 signaling [24]. An ongoing randomized placebo-controlled trial is currently evaluating NAC supplementation in PAP-treated patients with significant OSA (ClinicalTrials.gov NCT06311045), further supporting NAC as a testable adjunctive strategy rather than an established therapy. These findings highlight antioxidants and eGCX-stabilizing strategies as testable candidates. Whether HA and HS can serve as pharmacodynamic markers, and whether such approaches improve endothelial function or clinical outcomes in OSA, remains to be determined in prospective interventional studies.

In our study, several limitations warrant consideration. The clinical cohort was modest in size, recruited at a single center, and restricted to men, which limits generalizability and the precision of effect estimates. This restriction was chosen a priori to reduce documented biological variability related to hormonal cycle effects on circulating glycocalyx-related markers. However, the present findings might not be translatable to the relevant group of female OSA patients. Hence, investigating the relevance of these results for female patients with OSA is mandatory for a larger follow-up study. Although participants were recruited prospectively, the clinical biomarker analysis was effectively cross-sectional, precluding causal or temporal inference as to whether elevated HA and HS precede, follow, or merely accompany intermittent hypoxemia and OSA severity. Moreover, the study was not designed for formal biomarker validation or clinical risk stratification. Circulating HA and HS are not endothelium-specific and may also reflect extracellular matrix turnover or contributions from multiple (vascular) cell types. Future studies should therefore combine them with more source-informative approaches, such as endothelial-derived microparticles and direct heparanase/hyaluronidase activity measurements, to better define signal origin and mechanistic specificity. Finally, the in vitro intermittent hypoxia model captures an important component of OSA biology under controlled flow conditions, but it does not recapitulate the full complexity of human OSA, including sleep fragmentation, sympathetic activation and hypercapnia, and inter-individual variability in hypoxic burden.

**Fig. 6:**
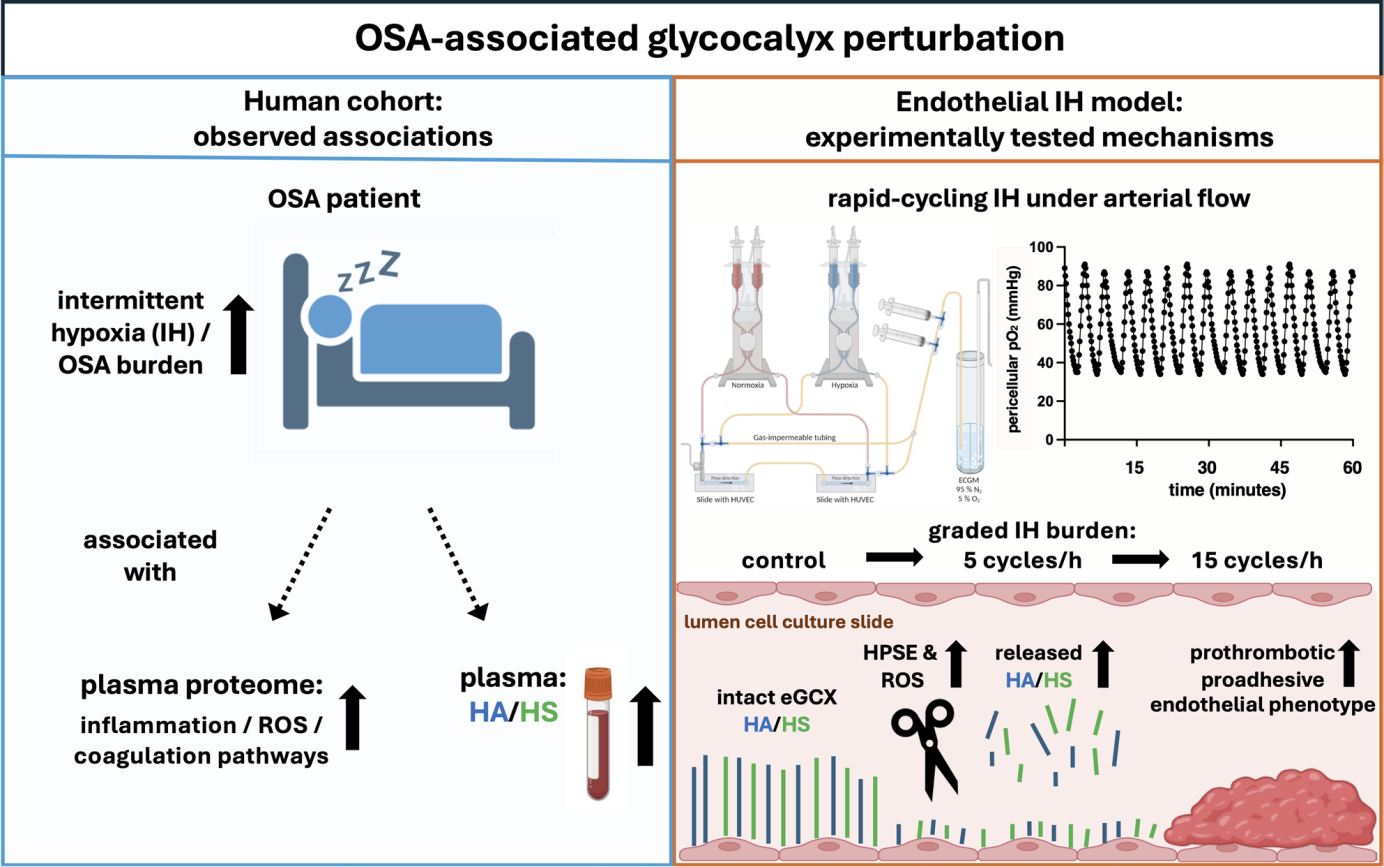
Schematic overview of clinical associations and experimentally tested endothelial mechanisms. The left panel summarizes findings from the human cohort, in which higher OSA burden and repetitive hypoxemia were associated with increased circulating HA and HS levels and with plasma proteomic signatures related to inflammation, ROS, and coagulation. The right panel summarizes experimentally tested mechanisms in the endothelial IH model under arterial flow, where graded intermittent hypoxia increased HPSE and ROS responses, promoted surface HA/HS loss and release, and induced a proadhesive and prothrombotic endothelial phenotype. Dotted arrows labeled “associated with” indicate clinical associations observed in the human cohort. HA, hyaluronan; HS, heparan sulfate; HPSE, heparanase; IH, intermittent hypoxia; OSA, obstructive sleep apnea; ROS, reactive oxygen species. Created with https://BioRender.com

Taken together, the integrated clinical, proteomic, and endothelial-model data support a role for glycocalyx perturbation as a biologically plausible component of OSA-associated vascular stress, as summarized schematically in Fig. 6.

## Conclusion

By pairing patient biomarkers with a mechanistically anchored IH model under physiological shear, we provide integrated evidence that OSA is associated with circulating markers consistent with glycocalyx perturbation in a cross-sectional clinical setting, while OSA-like IH induces oxidative–inflammatory stress, endothelial glycocalyx remodeling and proadhesive/thrombogenic endothelial changes in vitro. Of note, HA and HS are not endothelial-specific biomarkers and should currently be interpreted as candidate markers consistent with glycocalyx perturbation. They warrant evaluation in longitudinal and interventional studies, including cohorts with female participants and broader population diversity.

## Electronic supplementary material

Below is the link to the electronic supplementary material.

Supplementary Figure S1: Representative pericellular oxygen tension traces during graded intermittent hypoxia exposure. Representative pericellular oxygen tension traces during normoxic control conditions and rapid-cycling intermittent hypoxia at 5 or 15 cycles/h over 60 min. The 15 cycles/h condition represents the higher-burden IH paradigm used for most downstream endothelial assays, whereas 5 cycles/h represents the lower-burden IH condition. Traces illustrate rapid repetitive hypoxia–reoxygenation episodes under flow. IH, intermittent hypoxia.

Supplementary Figure S2: Multivariable-adjusted associations of circulating glycocalyx-related markers with OSA severity and hypoxemia. (A–C) Adjusted geometric means of plasma hyaluronan (HA), heparan sulfate (HS), and syndecan-1 (SDC-1) across non-OSA, mild, moderate, and severe OSA strata. Estimates were derived from parsimonious multivariable models adjusted for age, BMI, hypertension, hs-CRP, fasting glucose, and eGFR and back-transformed from the log10 scale. (D) Adjusted coefficient plot for continuous OSA and hypoxemia predictors. Points show the adjusted percent change in circulating HA or HS per +10 units of AHI, ODI3%, or hypoxic burden; error bars indicate 95% confidence intervals. Full model results and sensitivity analyses are provided in Supplementary Tables S3–S5. Data are shown as adjusted individual values with mean ± SEM; *p < 0.05, **p < 0.01, ***p < 0.001; ns, not significant.

Supplementary Figure S3: Stimulus-specific SDC-1 shedding and additional glycocalyx-remodeling mediators after IH. (A–D) TNF comparator experiments showing SDC-1 mRNA expression (A), endothelial surface SDC-1 expression measured by flow cytometry (B), soluble SDC-1 concentrations in cell-culture supernatants (C), and MMP2 concentrations in supernatants (D) after control conditions, IH, or TNF stimulation. (E, F) MMP2 mRNA expression and MMP2 protein concentrations in cell-culture supernatants after higher-burden IH compared with control. (G, H) ANGPT-2 mRNA expression and ANGPT-2 protein concentrations in cell-culture supernatants after IH compared with control. (I) HYAL1 mRNA expression after IH compared with control. (J–L) Thioredoxin reductase-1 (TrxR1/TXNRD1) expression assessed by qPCR (J), Western blot densitometry normalized to β-actin (K), and representative Western blot images (L). Data are mean ± SEM; *n* = 4–8 independent experiments, depending on assay; *p < 0.05, **p < 0.01; ns, not significant.

Supplementary Figure S4: Verification of enzymatic eGCX degradation and tissue factor induction after IH. (A, B) Surface HA (A) and HS (B) fluorescence intensity after enzymatic eGCX degradation by heparinase III and hyaluronidase compared with control. (C) Representative confocal images of HA (red), HS (green), and nuclei (blue) staining after control conditions and enzymatic eGCX degradation. Scale bars: 50 µm. (D) F3 mRNA expression measured by qPCR after higher-burden IH compared with control. (E) Endothelial tissue factor expression assessed by immunofluorescence after IH compared with control, with representative fluorescence images. Scale bars: 50 µm. Data are mean ± SEM; *n* = 3–6 independent experiments, depending on assay; *p < 0.05, **p < 0.01.

Supplementary Table S1: qPCR primers

Supplementary Table S2: Unadjusted correlation analyses

Supplementary Table S3: Multivariable-adjusted values from parsimonious models

Supplementary Table S4: ANCOVA results from parsimonious models

Supplementary Table S5: Sensitivity analyses

Supplementary Table S6: Differential plasma proteins, severe OSA versus non-OSA

Supplementary Table S7: Differential plasma proteins, mild versus severe OSA

Supplementary Table S8: Differential plasma proteins, non-OSA versus mild OSA

Supplementary Table S9: Over-representation analysis

Supplementary Table S10: Gene set enrichment analysis

Supplementary Table S11: Per-protein t-statistics and concordance analyses

## Data Availability

The mass spectrometry proteomics data have been deposited to the ProteomeXchange Consortium via the PRIDE partner repository under accession PXD073409. Source data underlying the figures are provided in the Supplementary/Additional files. De-identified clinical and polysomnography-derived data supporting the findings of this study are available from the corresponding author upon reasonable request, subject to institutional approvals and data protection regulations.

## Abbreviations

ACN: acetonitrile
AHI: apnea–hypopnea index
ANCOVA: analysis of covariance
BMI: body mass index
CPAP: continuous positive airway pressure
CVD: cardiovascular disease
DIA: data-independent acquisition
EC: endothelial cell(s)
EDTA: ethylenediaminetetraacetic acid
eGCX: endothelial glycocalyx
ELISA: enzyme-linked immunosorbent assay
FACS: fluorescence-activated cell sorting
FA: formic acid
FDR: false discovery rate
GSEA: gene set enrichment analysis
GCX: glycocalyx
GO: Gene Ontology
BP: biological process (Gene Ontology)
MF: molecular function (Gene Ontology)
HA: hyaluronan (hyaluronic acid)
HB: hypoxic burden
HPSE: heparanase
HS: heparan sulfate
HUVECs: human umbilical vein endothelial cells
IH: intermittent hypoxia
KEGG: Kyoto Encyclopedia of Genes and Genomes
MMP: matrix metalloproteinase
MS: mass spectrometry
NAC: N-acetylcysteine
NES: normalized enrichment score
NO: nitric oxide
NRF2: nuclear factor erythroid 2–related factor 2
ODI: oxygen desaturation index
ODI3: oxygen desaturation index (≥3% threshold)
OSA: obstructive sleep apnea
PCR: polymerase chain reaction
ROS: reactive oxygen species
SDC-1: syndecan-1
SEM: standard error of the mean
TNF: tumor necrosis factor-α
eNOS: endothelial nitric oxide synthase
