# Supplementary figures and images for: Endothelial glycocalyx perturbation in obstructive sleep apnea is associated with repetitive hypoxemia and immunothrombotic endothelial dysfunction
Source: J Transl Med. 2026 Jun 12;24:777. doi: 10.1186/s12967-026-08409-2 (PMC13277039; doi:10.1186/s12967-026-08409-2)

# Supplemental Figure S1

A

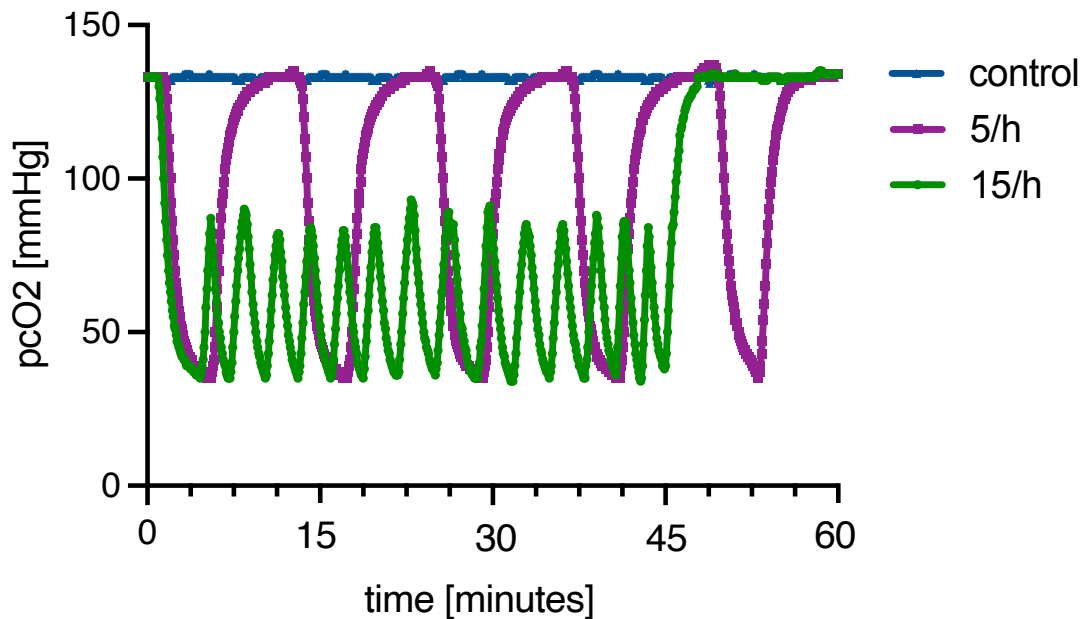

Supplement: Supplementary file 1 — Supplementary Figure S1: Representative pericellular oxygen tension traces during graded intermittent hypoxia exposure. Representative pericellular oxygen tension traces during normoxic control conditions and rapid-cycling intermittent hypoxia at 5 or 15 cycles/h over 60 min. The 15 cycles/h condition represents the higher-burden IH paradigm used for most downstream endothelial assays, whereas 5 cycles/h represents the lower-burden IH condition. Traces illustrate rapid repetitive hypoxia–reoxygenation episodes under flow. IH, intermittent hypoxia. [file 12967_2026_8409_MOESM1_ESM.pdf]

Supplemental Figure S2

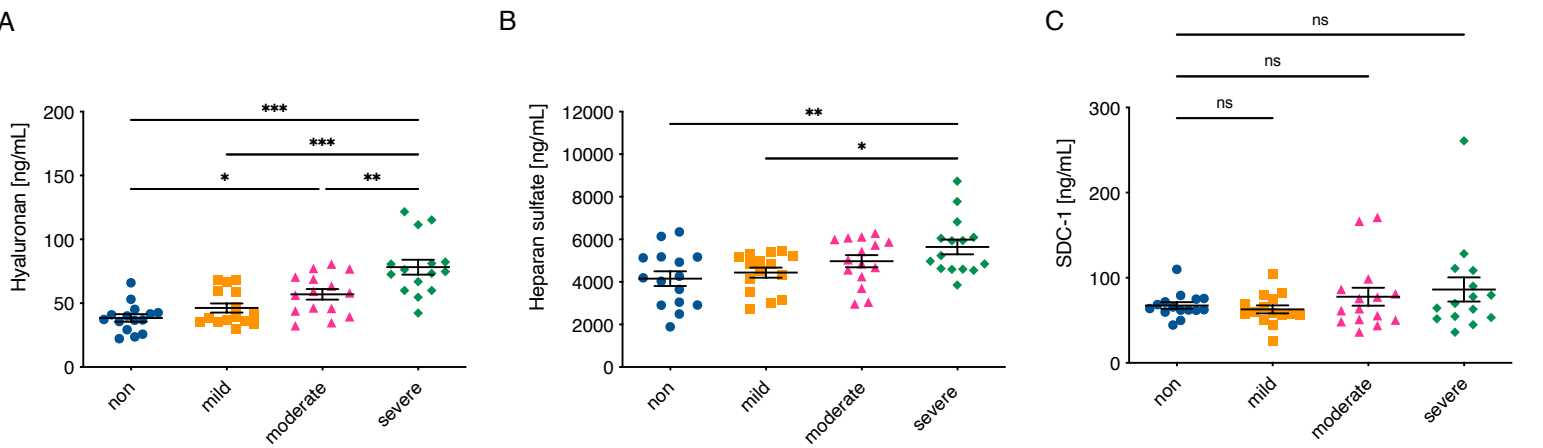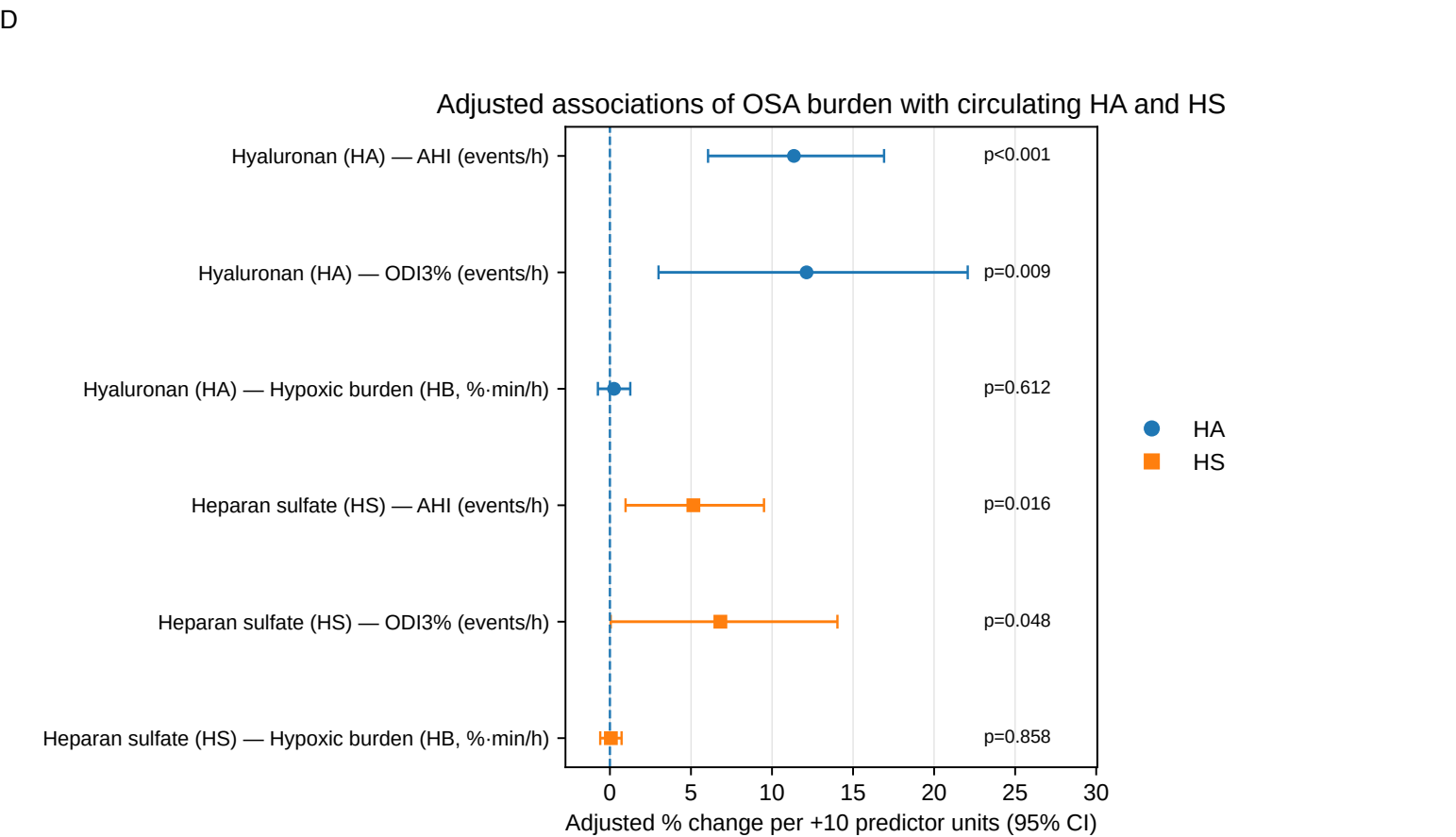

Supplement: Supplementary file 2 — Supplementary Figure S2: Multivariable-adjusted associations of circulating glycocalyx-related markers with OSA severity and hypoxemia. (A–C) Adjusted geometric means of plasma hyaluronan (HA), heparan sulfate (HS), and syndecan-1 (SDC-1) across non-OSA, mild, moderate, and severe OSA strata. Estimates were derived from parsimonious multivariable models adjusted for age, BMI, hypertension, hs-CRP, fasting glucose, and eGFR and back-transformed from the log10 scale. (D) Adjusted coefficient plot for continuous OSA and hypoxemia predictors. Points show the adjusted percent change in circulating HA or HS per +10 units of AHI, ODI3%, or hypoxic burden; error bars indicate 95% confidence intervals. Full model results and sensitivity analyses are provided in Supplementary Tables S3–S5. Data are shown as adjusted individual values with mean ± SEM; *p < 0.05, **p < 0.01, ***p < 0.001; ns, not significant. [file 12967_2026_8409_MOESM2_ESM.pdf]

Supplemental Figure S3

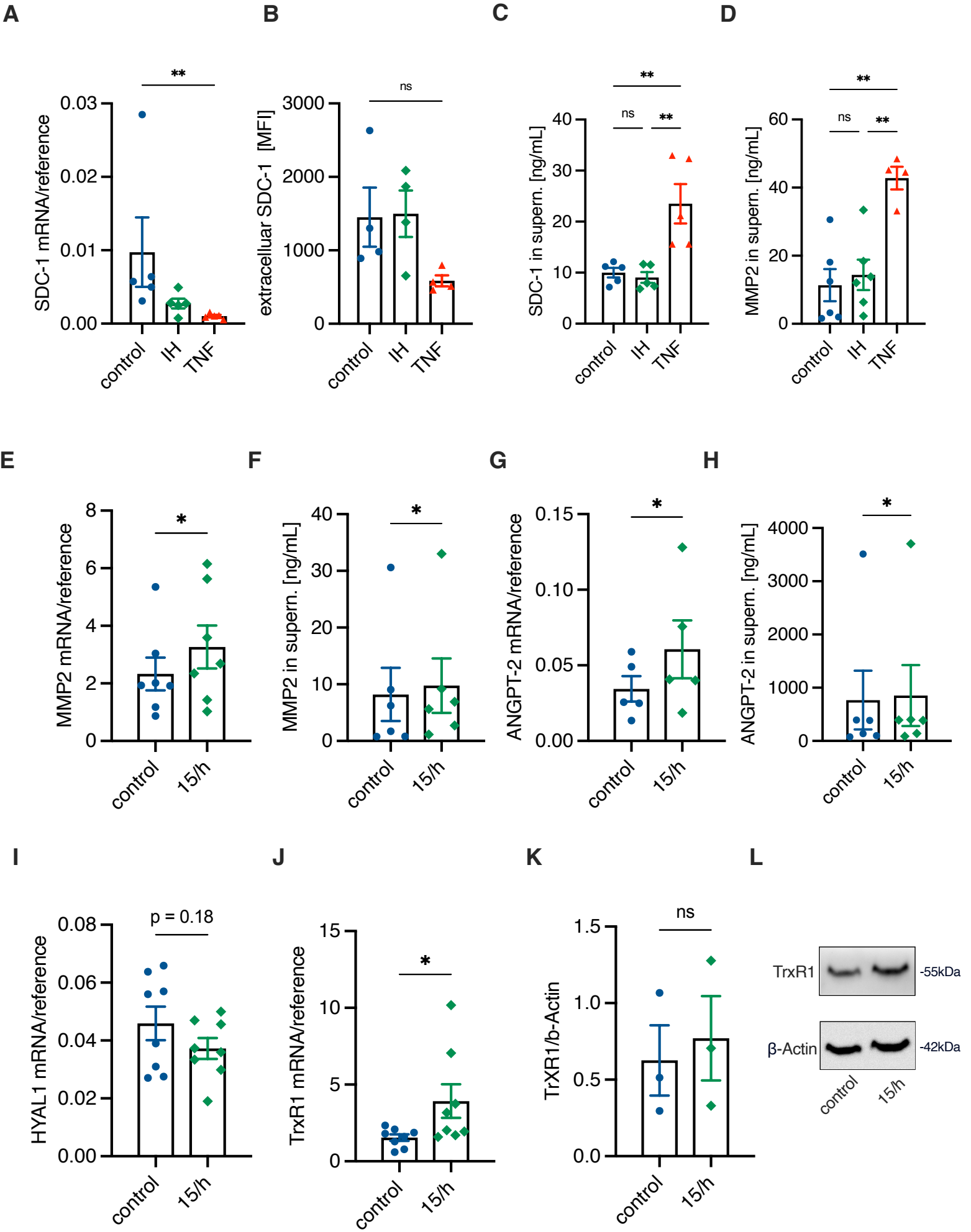

Supplement: Supplementary file 3 — Supplementary Figure S3: Stimulus-specific SDC-1 shedding and additional glycocalyx-remodeling mediators after IH. (A–D) TNF comparator experiments showing SDC-1 mRNA expression (A), endothelial surface SDC-1 expression measured by flow cytometry (B), soluble SDC-1 concentrations in cell-culture supernatants (C), and MMP2 concentrations in supernatants (D) after control conditions, IH, or TNF stimulation. (E, F) MMP2 mRNA expression and MMP2 protein concentrations in cell-culture supernatants after higher-burden IH compared with control. (G, H) ANGPT-2 mRNA expression and ANGPT-2 protein concentrations in cell-culture supernatants after IH compared with control. (I) HYAL1 mRNA expression after IH compared with control. (J–L) Thioredoxin reductase-1 (TrxR1/TXNRD1) expression assessed by qPCR (J), Western blot densitometry normalized to β-actin (K), and representative Western blot images (L). Data are mean ± SEM; n = 4–8 independent experiments, depending on assay; *p < 0.05, **p < 0.01; ns, not significant. [file 12967_2026_8409_MOESM3_ESM.pdf]

# Supplemental Figure S4

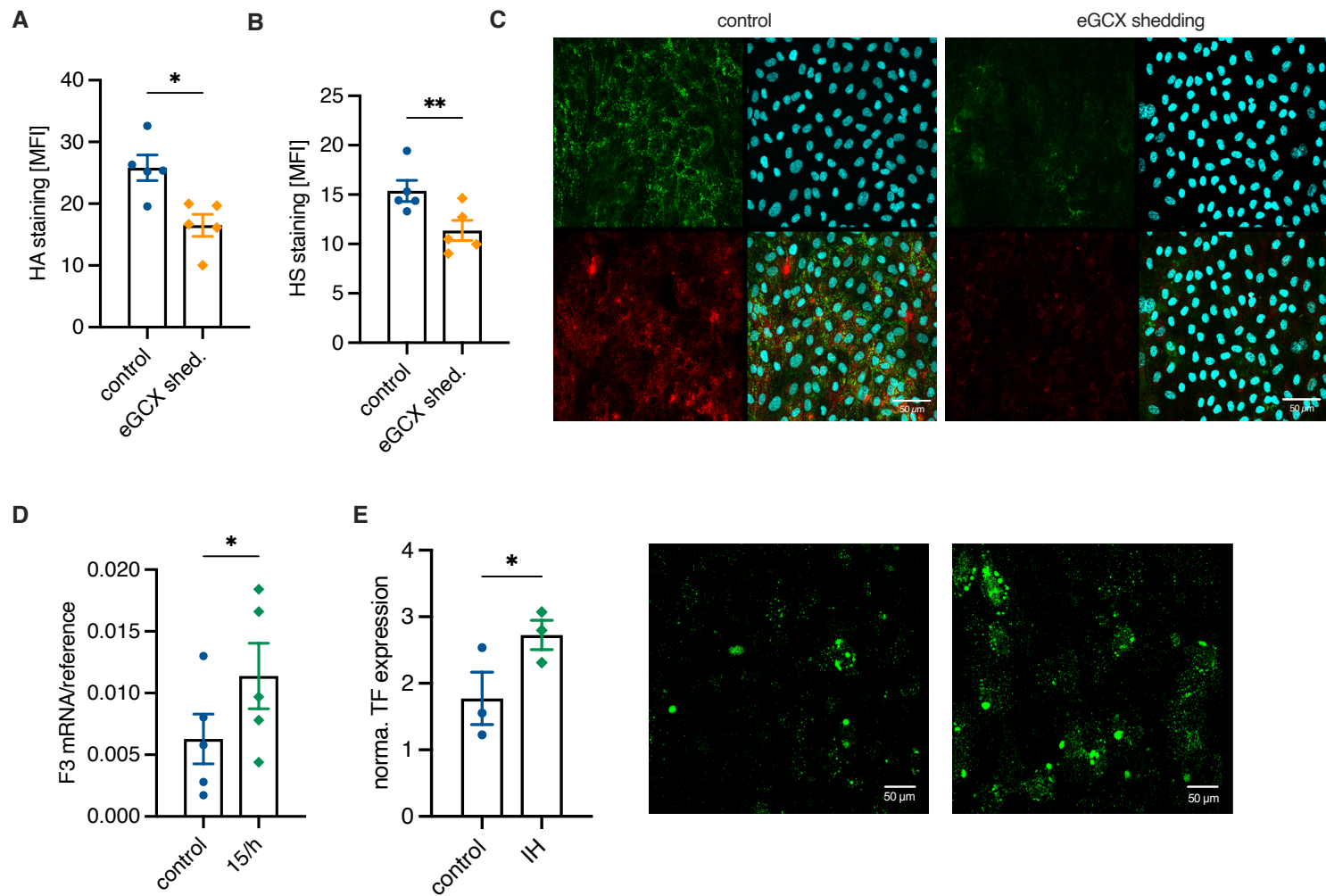

Supplement: Supplementary file 4 — Supplementary Figure S4: Verification of enzymatic eGCX degradation and tissue factor induction after IH. (A, B) Surface HA (A) and HS (B) fluorescence intensity after enzymatic eGCX degradation by heparinase III and hyaluronidase compared with control. (C) Representative confocal images of HA (red), HS (green), and nuclei (blue) staining after control conditions and enzymatic eGCX degradation. Scale bars: 50 µm. (D) F3 mRNA expression measured by qPCR after higher-burden IH compared with control. (E) Endothelial tissue factor expression assessed by immunofluorescence after IH compared with control, with representative fluorescence images. Scale bars: 50 µm. Data are mean ± SEM; n = 3–6 independent experiments, depending on assay; *p < 0.05, **p < 0.01. [file 12967_2026_8409_MOESM4_ESM.pdf]
