# Supplementary material for: Endothelial glycocalyx perturbation in obstructive sleep apnea is associated with repetitive hypoxemia and immunothrombotic endothelial dysfunction
Source: J Transl Med. 2026 Jun 12;24:777. doi: 10.1186/s12967-026-08409-2 (PMC13277039; doi:10.1186/s12967-026-08409-2)
Supplement: Supplementary file 5 — Supplementary Table S1: qPCR primers [file 12967_2026_8409_MOESM5_ESM.docx]

**Supplementary table S1, qPCR primers**

| **Transcript name** | **Sequence** | **Source** | **Persistent ID / URL** |
| --- | --- | --- | --- |
| NRF2 | F: GAGACAGGTGAATTTCTCCCAAT; R: TTTGGGAATGTGGGCAAC; Probe: UPL #26 | Roche ProbeFinder / UPL |  |
| MMP2 | F: CCCCAAAACGACAAAGAG; R: CTTCAGCACAAACAGGTTGC; Probe: UPL #43 | Roche ProbeFinder / UPL |  |
| TXNRD1 | F: CAATTGGAATCCACCCTGTC; R: CCACACTGGGGCTTAACCT; Probe: UPL #64 | Roche ProbeFinder / UPL |  |
| NOS3 (eNOS) | F: GATCCCCCAGAACTCTTCCT; R: CAGGGCTGCAAACCACTC; Probe: UPL #1 | Roche ProbeFinder / UPL |  |
| ANGPT2 | F: ATCAGCCAACCAGGAAATGA; R: AGGACCACATGCATCAAACC; Probe: UPL #58 | Roche ProbeFinder / UPL |  |
| HPSE | F: CACCAGGGATCTGGGTTCT; R: AGAATGAAGCCAGCGCTAAA; Probe: UPL #53 | Roche ProbeFinder / UPL |  |
| VWF(PrimePCR™ Probe Assay) | Commercial qPCR probe assay | Bio-Rad | Unique assay ID:  qHsaCEP0050592 |
| F3 (PrimePCR™ Probe Assay) | Commercial qPCR probe assay | Bio-Rad | Unique assay ID:  qHsaCEP0039176 |
| GAPDH (PrimePCR™ Probe Assay) | Commercial qPCR probe assay | Bio-Rad | Unique assay ID:  qHsaCEP0041396 |
| TBP (PrimePCR™ Probe Assay) | Commercial qPCR probe assay | Bio-Rad | Unique assay ID:  qHsaCIP0036255 |
